# Huanglian Jiedu decoction alleviates ischemia‐induced cerebral injury in rats by mitigating NET formation and activiting GABAergic synapses

**DOI:** 10.1111/jcmm.18528

**Published:** 2024-08-04

**Authors:** Youxiang Cui, Mingyue Cui, Leilei Wang, Ning Wang, Yao Chen, Shuquan Lv, Limin Zhang, Congai Chen, Yanwen Yang, Feng Wang, Lichun Wang, Huantian Cui

**Affiliations:** ^1^ Key Laboratory of Neurological Rehabilitation Cangzhou Hospital of Integrated Traditional Chinese Medicine and Western Medicine Cangzhou China; ^2^ First School of Clinical Medicine Yunnan University of Chinese Medicine Kunming China; ^3^ Beijing University of Chinese Medicine Beijing China

**Keywords:** GABAergic synapses, Huanglian Jiedu decoction, ischemia‐induced cerebral injury, neutrophil extracellular trap formation, traditional Chinese medicine, transcriptomics

## Abstract

Huanglian Jiedu decoction (HLJD) has been used to treat ischemic stroke in clinic. However, the detailed protective mechanisms of HLJD on ischemic stroke have yet to be elucidated. The aim of this study is to elucidate the underlying pharmacological mechanisms of HLJD based on the inhibition of neuroinflammation and the amelioration of nerve cell damage. A middle cerebral artery occlusion reperfusion (MCAO/R) model was established in rats and received HLJD treatment. Effects of HLJD on neurological function was assessed based on Bederson's score, postural reflex test and asymmetry score. 2, 3, 5‐Triphenyltetrazolium chloride (TTC) staining, Hematein and eosin (HE) and Nissl staining were used to observe the pathological changes in brain. Then, transcriptomics was used to screen the differential genes in brain tissue in MCAO/R model rats following HLJD intervention. Subsequently, the effects of HLJD on neutrophil extracellular trap (NET) formation‐related neuroinflammation, gamma‐aminobutyric acid (GABA)ergic synapse activation, nerve cell damage and proliferation were validated using immunofluorescence, western blot and enzyme‐linked immunosorbent assay (ELISA). Our results showed that HLJD intervention reduced the Bederson's score, postural reflex test score and asymmetry score in MCAO/R model rats. Pathological staining indicated that HLJD treatment decreased the cerebral infarction area, mitigated neuronal damage and increased the numbers of Nissl bodies. Transcriptomics suggested that HLJD affected 435 genes in MCAO/R rats. Among them, several genes involving in NET formation and GABAergic synapses pathways were dysregulated. Subsequent experimental validation showed that HLJD reduced the MPO^+^CitH3^+^ positive expression area, reduced the protein expression of PAD4, p‐P38/P38, p‐ERK/ERK and decreased the levels of IL‐1β, IL‐6 and TNF‐α, reversed the increase of Iba1^+^TLR4^+^, Iba1^+^p65^+^ and Iba1^+^NLRP3^+^ positive expression area in brain. Moreover, HLJD increased GABA levels, elevated the protein expression of GABRG1 and GAT3, decreased the TUNEL positive expression area and increased the Ki67 positive expression area in brain. HLJD intervention exerts a multifaceted positive impact on ischemia‐induced cerebral injury in MCAO/R rats. This intervention effectively inhibits neuroinflammation by mitigating NET formation, and concurrently improves nerve cell damage and fosters nerve cell proliferation through activating GABAergic synapses.

## 1 INTRODUCTION

Ischemic stroke ranks among the most prevalent cerebrovascular diseases encountered in clinical practice. Its primary pathological manifestations involve brain edema and necrosis at the lesion site, which result in brain tissue atrophy, structural damage and cortical thinning, posing a grave threat to the patient's life.^1^ The escalating incidence and mortality rates of ischemic stroke, coupled with the elevated risk of enduring cognitive dysfunction and dementia following a stroke, have led to a substantial upsurge in the socioeconomic burden.^2^, ^3^ The constrained treatment window for ischemic stroke presents a formidable obstacle to the development of efficacious therapeutic approaches.^4^, ^5^ Currently, the principal treatment modalities encompass thrombolytic therapy,^6^, ^7^ anticoagulant therapy,^8^ endovascular therapy,^9^, ^10^ and neuroprotective therapy.^11^, ^12^ Neuroprotective therapy assumes a critical role in the management of ischemic stroke, and when compared to thrombolytic therapy with its rigid time constraints and potential severe complications, early implementation of neuroprotective therapy in ischemic stroke yields superior outcomes.^13^ Nevertheless, compelling evidence is currently lacking for the neuroprotective properties of most drugs in the treatment of ischemic stroke, necessitating the urgent quest for effective therapeutic strategies to combat ischemia‐induced cerebral injury.

Neuroinflammation constitutes a fundamental component of the pathophysiology of ischemic stroke, and its impact extends throughout the post‐stroke period.^14^ In response to ischemia‐induced cerebral injury, the nervous system promptly triggers inflammatory responses.^15^ This rapid activation of microglia and astrocytes stimulates the production of pro‐inflammatory cytokines^16^, ^17^ and activates key signalling pathways including neutrophil extracellular traps (NETs),^18^ toll‐like receptor 4 (TLR4),^19^ nuclear factor‐kappaB (NF‐κB),^20^ and NOD‐like receptor thermal protein domain associated protein 3 (NLRP3),^21^ all of which contribute to neuronal damage. A plethora of studies has substantiated that the inhibition of inflammation can effectively ameliorate ischemia‐induced cerebral injury.^22^, ^23^

The inhibition of inflammatory responses represents a pivotal mechanism in the traditional Chinese medicine approach to the treatment of ischemic stroke. Taohong Siwu decoction, for instance, can attenuate ischemia‐induced cerebral injury in middle cerebral artery occlusion reperfusion (MCAO/R) rats by reducing the levels of pro‐inflammatory cytokines such as tumour necrosis factor alpha (TNF‐α), interleukin 6 (IL‐6) and interleukin‐1beta (IL‐1β).^24^ Tongxinluo exhibits the capacity to suppress the caspase‐11/gasdermin D (GSDMD) pathway‐mediated pyroptosis of astrocytes, thus mitigating the associated neuroinflammatory response.^25^ Gualou Guizhi decoction, by inhibiting NF‐κB activation, can curtail the release of pro‐inflammatory factors in MCAO/R rats.^26^ Clinical investigations have further revealed that traditional Chinese medicine confers distinct advantages in improving ischemia‐induced cerebral injury in patients. Wendan decoction^27^ and Xueshuantong injection^28^ are effective in enhancing the recovery of neurological function in post‐stroke patients. Qizhi Tongluo capsule can promote the restoration of lower limb motor function in post‐stroke patients.^29^ Dengzhan Shengmai capsule, commonly employed to alleviate neurological injury after a stroke, also demonstrates efficacy in preventing recurrent strokes.^30^

Huanglian Jiedu decoction (HLJD), composed of *Coptis chinensis* Franch., *Scutellaria baicalensis* Georgi, *Phellodendron amurense* Rupr. and *Gardenia jasminoides* J.Ellis, has exhibited therapeutic potential in the MCAO/R model. A study has demonstrated that HLJD can mitigate thrombosis in the MCAO/R model,^31^ while another study has established its capacity to ameliorate ischemia‐induced cerebral injury in the MCAO/R model.^32^ Notably, the anti‐inflammatory properties of HLJD have been extensively documented.^33^, ^34^ However, the detailed protective mechanisms of HLJD on ischemic stroke have yet to be elucidated. Thus, we first established an MCAO/R rat model to explore the therapeutic potential of HLJD in mitigating ischemia‐induced cerebral injury. Building upon this foundation, we employed transcriptomics to delve into the impact of HLJD intervention on gene expression in MCAO/R rats. Interestingly, our findings revealed that HLJD downregulates a multitude of genes associated with the inflammatory response. Subsequently, we validated the effect of HLJD on the inflammatory response‐related pathway (NET formation) in MCAO/R rats. Furthermore, we conducted investigations to confirm the effects of HLJD on gamma‐aminobutyric acid (GABA) transmission and neuronal injury repair, drawing on the insights gained from the transcriptomics results in MCAO/R rats.

## 2 METHODS

### 2.1 Animals and Reagents

Male Sprague Dawley (SD) rats aged 6–8 weeks, weighing 230 ± 10 g, were obtained from SPF (Beijing) Biotechnology Co., Ltd. (Animal Production Licence No.: SCXK (Beijing) 2019–0010, Beijing, China). Animal experiments were conducted within a Specific Pathogen‐Free (SPF)‐grade facility, with five rats per cage, provided with standard feed and unrestricted access to food and water. All animal experiments were conducted in compliance with the ‘Guidelines for the Care and Use of Laboratory Animals’ issued by the National Institutes of Health and were approved by the Ethical Review Committee of Animal Experiments in Yunnan University of Chinese Medicine (Approval No.: R‐062023LH265), dated March 07, 2023. Detailed information regarding the materials and kits utilized in this experiment is available in the supplementary materials.

### 2.2 Preparation of HLJD

In accordance with the HLJD prescription, the following quantities of ingredients were weighed: 6 g of *Coptis chinensis* Franch., 6 g of *Scutellaria baicalensis* Georgi, 6 g of *Phellodendron amurense* Rupr. and 9 g of *Gardenia jasminoides* J.Ellis. Subsequently, eight times the volume of water was added for a 30‐minute soaking period, followed by boiling over high heat and simmering over low heat for an additional 30 min. The initial batch of medicinal liquid was filtered and set aside. The decoction process was repeated twice, and the medicinal liquids from the two batches were combined. The mixture was concentrated into 1 g crude herb/mL, and stored at 4°C. The main active components berberine, baicalin and geniposide in HLJD were detected by high performance liquid chromatography (HPLC), as shown in the supplementary materials.

### 2.3 Model for MCAO/R rats

The rats were first raised for a 7‐day period for adaptation. They were then fasted for food (not for water) for 12 h before modelling. The MCAO/R model was created according to a previously reported method.^35^ Briefly, the external carotid artery (ECA), internal carotid artery (ICA) and common carotid artery (CCA) were exposed after the rats were anaesthetised by intraperitoneal injection of sodium pentobarbital (50 mg/kg). A thread was inserted into the middle cerebral artery through the CCA, ECA and ICA. The thread was removed after 2 h. Rats in sham group underwent the same process except for ligation and thread insertion.

### 2.4 Grouping and drug administration

We randomly allocated 90 SD rats into six groups using a random number table method: Sham group, MCAO/R group, Ginaton group, low‐dose HLJD group (HLJD‐L), middle‐dose HLJD group (HLJD‐M) and high‐dose HLJD group (HLJD‐H). All groups, except the Sham group, underwent the establishment of MCAO/R models. Following model induction, the Sham group and the MCAO/R group received oral gavage with 1 mL/100 g of normal saline, while the Ginaton group received oral gavage with ginaton at a dose of 21.6 mg/kg^32^. The HLJD‐L, −M and ‐H groups were orally administered HLJD at doses of 1.22, 2.43 and 4.86 g crude herb/kg, respectively. Each group received oral gavage every 12 h for a continuous period of 5 days. The doses of HLJD were extrapolated from human doses to animal equivalent doses based on body surface area, with the middle dose representing the equivalent dose. After 5 days of drug intervention, rats were sacrificed and brain samples were collected.

### 2.5 Neurological function evaluation

We assessed the neurological function of the rats using Bederson's score,^36^, ^37^ the postural reflex test,^38^ and the asymmetry score.^39^ In essence, Bederson's score primarily observed the extent of spontaneous rotation of the rat's body to the right side, while the postural reflex test predominantly examined the degree of extension of the right forelimb to the ventral side of the rat in an inverted hanging state. The asymmetry score chiefly recorded the frequency of contact between the left and right forelimbs with the edge of the table, individually, in an inverted hanging position. Please refer to supplementary Table S1 for specific evaluation criteria.

### 2.6 2, 3, 5‐Triphenyltetrazolium Chloride (TTC) Staining

As per established literature protocols, we assessed the infarct size of the brain.^40^ In brief, the rat brain was serially sectioned into six coronal slices, which were then subjected to staining with 2% TTC for 15 min in a light‐protected environment at 37.0°C. Subsequently, the cerebral infarction area of each slice was photographed and quantified using Image J (NIH, Bethesda, MD, USA).

### 2.7 Pathological Staining

After fixation (4% paraformaldehyde), dehydration (with series ethanol) and paraffin embedding, the rat brain tissues were cut into 5 μm‐thick slices for staining with HE,^41^ Nissl,^42^ and terminal deoxynucleotidyl transferase (TdT) dUTP Nick‐End Labelling (TUNEL).^43^ The HE and Nissl stained slices were photographed under the CX22 optical microscope (Olympus, Tokyo, Japan). The TUNEL stained slices were observed via an ECLIPSE C1 fluorescence microscope (NIKON, Tokyo, Japan). The severity of pathological damage as indicated by HE staining was evaluated using the denatured cell index (DCI), which represents the number ratio of denatured cells to total cells.^44^ Image Pro Plus 6.0 (Media Cybernetics, Silver Springs, MD, USA) was used to measure the Nissl bodies and the positive areas of TUNEL.

### 2.8 Transcriptomics assay

50 mg of brain tissue from each group of rats was added to a lysis buffer. mRNA with polyA was enriched using Oligo (dT) magnetic beads, followed by ion fragmentation. Using the fragmented mRNA as a template and random oligonucleotides as primers, the first and second strands of the cDNA were synthesized in a reverse transcriptase system. After purification, a strand of cDNA of about 400 bp was selected, amplified by PCR and purified, from which a library was obtained. The library was then quality tested using qRT‐PCR. Illumina sequencing was performed to generate raw data in fastq format and to perform quality control. HISAT2 (version: 2.0.5) and StringTie (version: 1.3.3b) were used for sequence alignments, and new gene predictions, respectively. Gene expression levels were quantified via Feature Counts (version: 1.5.0‐p3), and their expression differences were analysed via EdgeR (version: 3.22.5). Kyoto Encyclopedia of Genes and Genomes (KEGG) enrichment for differentially expressed genes was carried out using ClusterProfiler (version: 3.8.1).

### 2.9 Immunofluorescence assay

We began by deparaffinizing, hydrating and repairing the antigen in the rat brain tissue sections. Subsequently, the sections were subjected to incubation with a 3% hydrogen peroxide solution at 25.0°C. Following this, a 20% normal goat serum was applied at 4.0°C for 1 h, and then the sections were exposed to the following primary antibodies: mouse anti‐myeloperoxidase (MPO) (1/50) and rabbit anti‐citrullinated histone H3 (CitH3) (1/100), rabbit anti‐ionized calcium binding adaptor molecule 1 (Iba1) (1/100) and mouse anti‐TLR4 (1/100), rabbit anti‐Iba1 (1/100) and mouse anti‐p65 (1/400) and mouse anti‐Iba1 (1/100) and rabbit anti‐NLRP3 (1/300), rabbit anti‐Ki67 (1/250) at 4.0°C for 12 h. Subsequent to washing, the sections were treated with fluorescein‐conjugated secondary antibodies at 25.0°C for 1 h, and the cell nuclei were stained with DAPI solution and an anti‐fluorescent quencher. Fluorescent images were captured using a fluorescence microscope, and the areas displaying positive expression were quantified using Image Pro Plus 6.0.

### 2.10 Enzyme‐linked immunosorbent assay (ELISA) assay

The levels of IL‐1β, IL‐6, TNF‐α and GABA in brain tissue were determined through ELISA. The specific procedural steps were executed in accordance with the guidelines provided by the reagent kit instructions.

### 2.11 Western blot assay

Rat brain homogenates (100 mg) were lysed in the RIPA lysis buffer and the total protein was extracted. After the denatured proteins were isolated through SDS‐PAGE, PVDF membranes became a new carrier. Then, membranes were blocked for 1 h with 5% skim milk. The primary antibodies peptidylarginine deiminase 4 (PAD4), phosphor (p)‐P38, P38, p‐extracellular signal‐regulated kinase (ERK), ERK, gamma‐amino butyric acid type A receptor gamma1 subunit (GABRG1), GABA transporter 3 (GAT3) and β‐actin were incubated at 4.0°C for 12 h, with a 1/1000 dilution ratio of primary antibodies. The secondary antibody was incubated at 25.0°C for 1 h. The blot was developed with an enhanced chemiluminescence (ECL) kit, and quantitative analysis was performed using Image J.

### 2.12 Statistical analysis

All data were statistically analysed using the SPSS Pro platform (Zhongyan, Shanghai, China), and are presented as means ± SD. The Shapiro–Wilk test was used to check normality of the data. Tukey's post‐tests and one‐way ANOVA were used to compare group differences. *p* < 0.05 indicates statistical significance.

## 3 RESULTS

### 3.1 HLJD exhibited obvious therapeutic effect on MCAO/R rats

**FIGURE 1:**
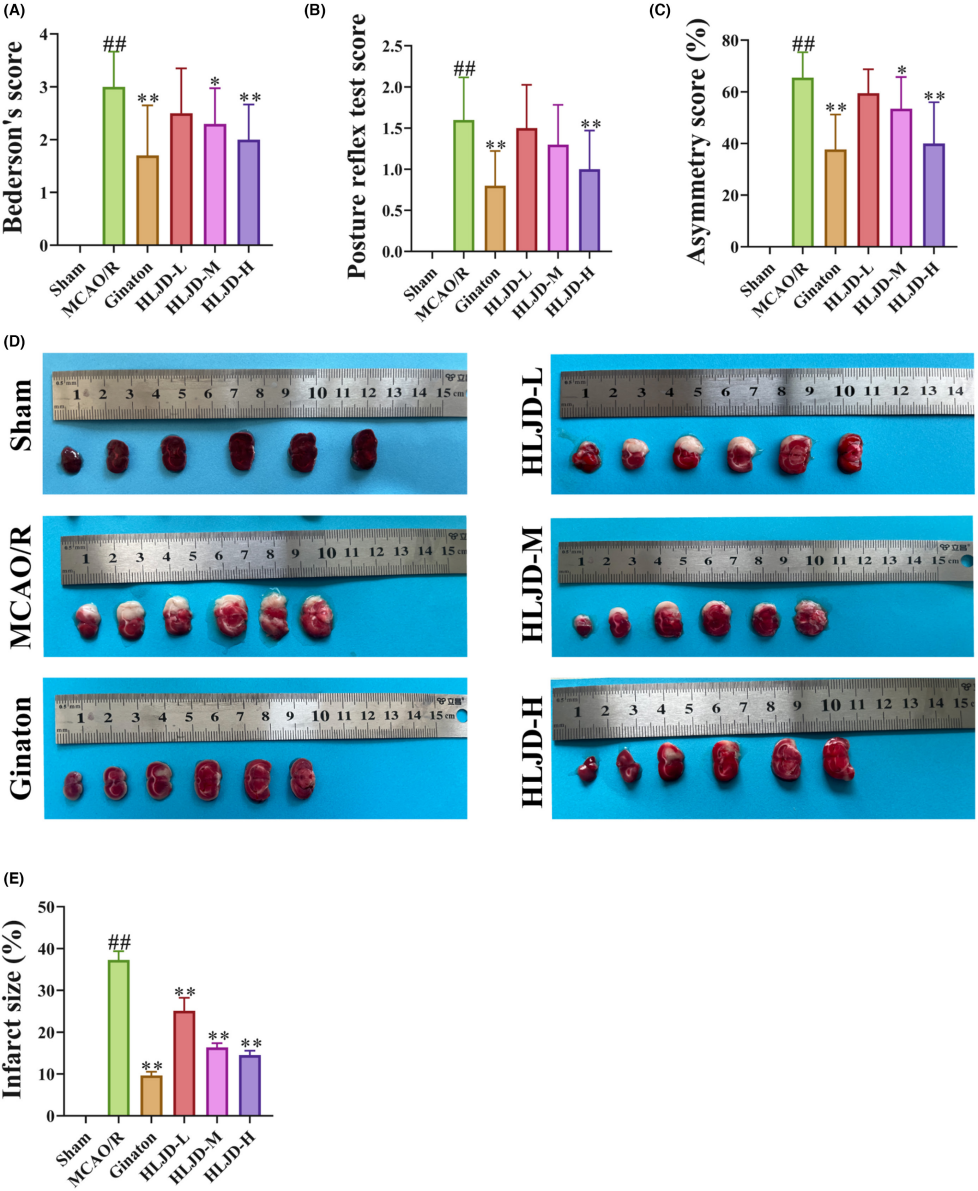
Huanglian Jiedu decoction (HLJD) promoted the recovery of neurological function and reduced the infarct area in middle cerebral artery occlusion reperfusion (MCAO/R) rats. MCAO/R rats received HLJD treatment for 5 days. Meanwhile, Ginaton was used as positive control. Bederson's score, posture reflex test score and asymmetry score were used to assess neurological function, 2, 3, 5‐Triphenyltetrazolium chloride (TTC) staining was used to observe infarct areas. (A–E) Results showed that HLJD treatment reduced Bederson's score (A), posture reflex test score (B) and asymmetry score (C); and decreased infarct areas (D, E). Data were presented as the mean ± SD, *n* = 15 for the neurological score and *n* = 6 for TTC staining. #*p* < 0.05, ##*p* < 0.01 compared to the sham group; *p < 0.05, **p < 0.01 compared to the MCAO/R group.

To assess the recovery of neurological function in MCAO/R rats, we initially conducted Bederson's score, the postural reflex test and the asymmetry score (Figure 1A–C). The results from Bederson's score and the postural reflex test demonstrated that the Sham group did not exhibit significant neurological deficits, whereas the MCAO/R rats displayed a significant increase in neurological deficit scores. Importantly, HLJD intervention significantly ameliorated these deficits. Concurrently, HLJD significantly reduced the asymmetry score, suggesting that it may enhance forepaw activity and promote the recovery of neurological function in MCAO/R rats. Furthermore, TTC staining revealed a significant increase in the cerebral infarction area in the MCAO/R group compared to the Sham group, which significantly decreased following HLJD intervention (Figure 1D,E).

**FIGURE 2:**
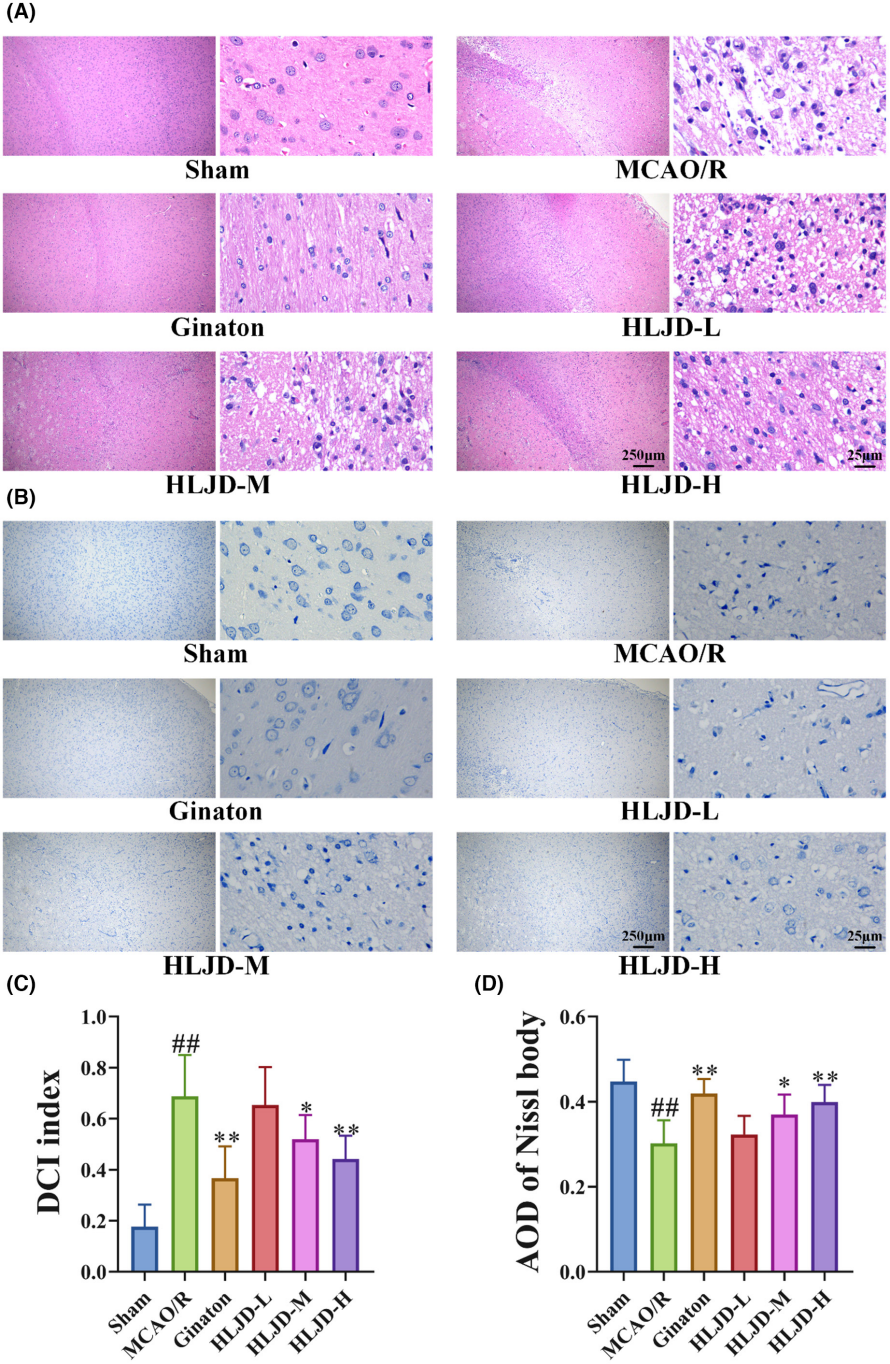
Huanglian Jiedu decoction (HLJD) ameliorated pathological changes in the brain in MCAO/R rats. (A, C) Hematein and eosin (HE) staining showed that HLJD treatment reduced the denatured cell index index in brain. (B, D) Nissl staining showed that HLJD treatment reduced the average optical density (AOD) of Nissl body in brain (Magnification: 40× and 400×, respectively). *n* = 3 per group.

To further evaluate the impact of HLJD intervention on pathological changes in the brain tissue of MCAO/R rats, we performed HE and Nissl staining. HE staining revealed that neurons in the Sham group displayed regular morphology, clear nucleoli, abundant cytoplasm and well‐arranged cells. In contrast, the MCAO/R group exhibited glial cell proliferation, disordered neuronal arrangement, widened intercellular spaces, nuclear condensation and the presence of reticular lesions. However, HLJD intervention significantly mitigated neuronal damage (Figure 2A,C). Nissl staining indicated a significant decrease in the number of Nissl bodies in the MCAO/R group compared to the Sham group, while HLJD intervention reversed this outcome (Figure 2B,D). Notably, among the different doses of HLJD, high‐dose HLJD demonstrated the most favourable outcomes in terms of neurofunctional recovery, reduction of cerebral infarction area and recovery from neuronal damage. Consequently, we proceeded with the analysis using high‐dose HLJD.

### 3.2 Differentially expressed genes in brain the following HLJD treatment

**FIGURE 3:**
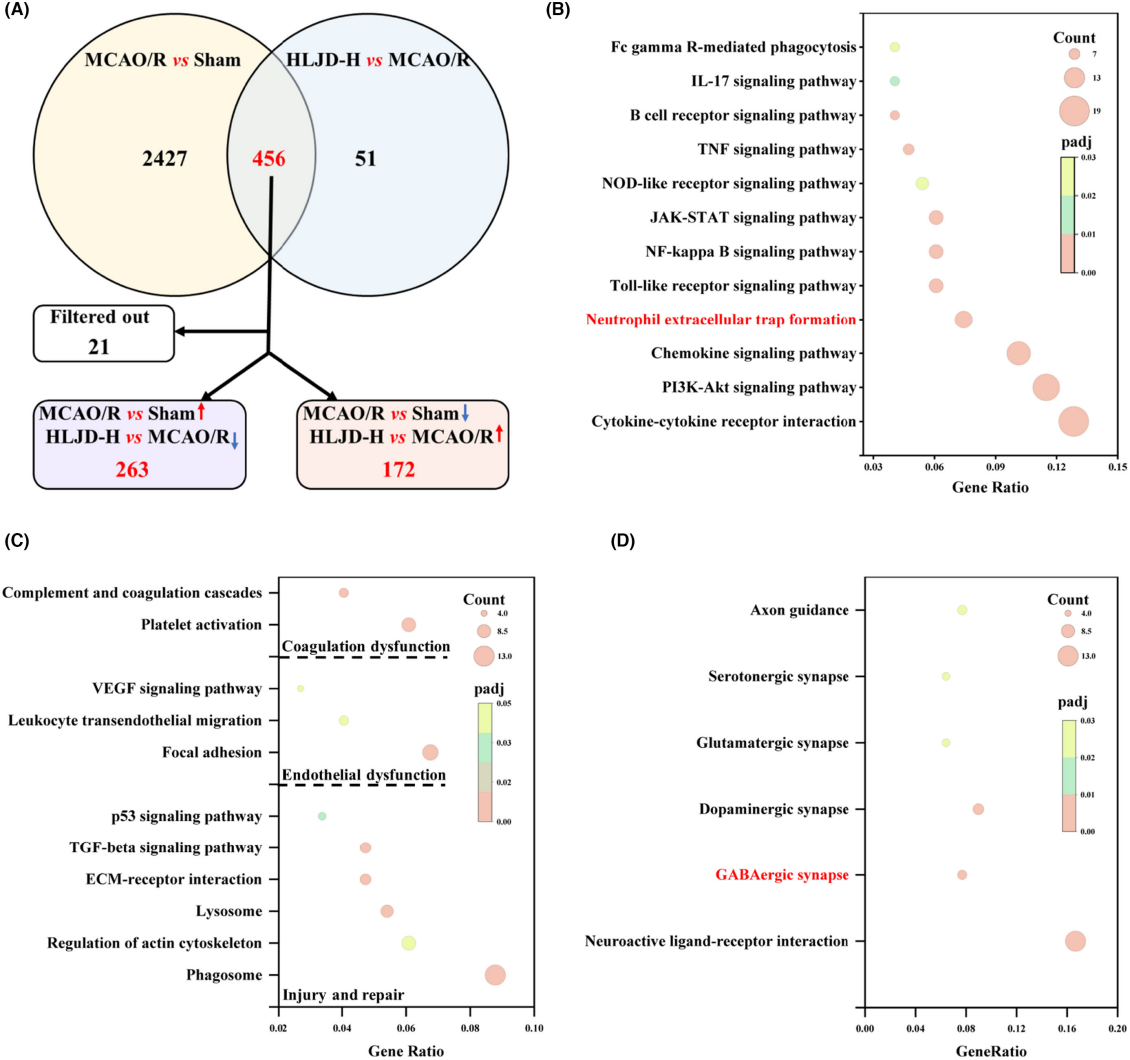
Differentially expressed genes in brain following Huanglian Jiedu decoction (HLJD) treatment were screened using transcriptomics. Brain tissues in the Sham, MCAO/R and HLJD‐H groups were used for transcriptomics analysis. (A) The protocol for screening of differentially regulated genes was visualized by a Venn diagram. (B–D) Kyoto Encyclopedia of Genes and Genomes (KEGG) results showed that down‐regulated genes by HLJD treatment were mainly related to inflammation (B), endothelial dysfunction (C), coagulation dysfunction (C), injury and repair (C) pathways and up‐regulated genes by HLJD treatment were mainly related to nerve conduction (D). *n* = 6 per group.

We conducted differential gene analysis comparing the MCAO/R group versus Sham group and the HLJD‐H group versus MCAO/R group, adhering to the criteria of |Log2(FoldChange)| ≥ 1 and padj ≤0.05. In this analysis, 2883 differential genes were identified in the MCAO/R group versus Sham group, and 507 differential genes were detected in the HLJD‐H group versus MCAO/R group, with 456 genes shared between the two groups. We further scrutinized these 456 genes to identify those that were up‐regulated in the MCAO/R group versus Sham group and down‐regulated in the HLJD‐H group versus MCAO/R group, as well as genes that exhibited the opposite pattern, as potential candidates for HLJD treatment of ischemic stroke. Our findings revealed that HLJD intervention down‐regulated 263 genes and up‐regulated 172 genes (Figure 3A). Please refer to the supplementary materials for specific gene information.

Subsequently, we employed KEGG analysis to investigate the pathways associated with these differential genes. The results indicated that the pathways linked to down‐regulated genes by HLJD were primarily related to inflammation, injury and repair, coagulation disorders and endothelial dysfunction (Figure 3B,C). And, the pathways associated with up‐regulated genes by HLJD were primarily connected to neural transmission (Figure 3D). Interestingly, among the down‐regulated gene‐related pathways, NET formation (KEGG_ID: rno04613) was identified as being relevant to inflammation,^45^ injury and repair,^18^ coagulation disorders,^46^ and endothelial dysfunction.^47^ Among the up‐regulated gene‐related pathways, GABAergic synapse (KEGG_ID: rno04727) was found to be involved in neural transmission.^48^ Thus, we proceeded to validate the effects of HLJD on NET formation and GABAergic synapse, as well as their associated factors.

### 3.3 HLJD mitigated NET‐related neuroinflammation

**FIGURE 4:**
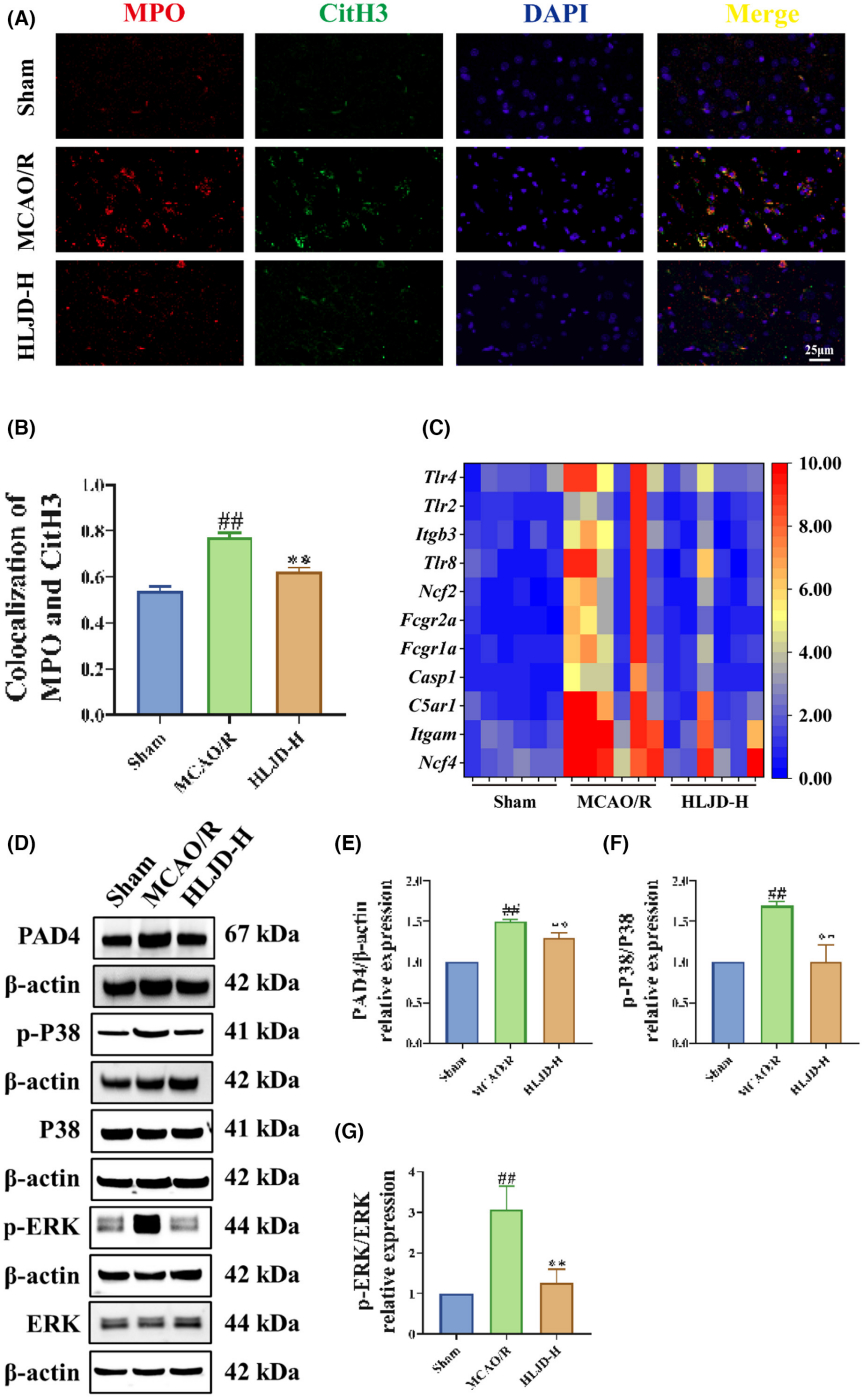
Huanglian Jiedu decoction (HLJD) treatment inhibited neutrophil extracellular trap (NET) formation in MCAO/R rats. (A, B) Immunofluorescence results showed that HLJD treatment reduced myeloperoxidase (MPO)^+^ citrullinated histone H3 (CitH3)^+^ positive expression area (Magnification: 400×). (C) Differentially expressed genes involving in NET formation obtained from transcriptomics were visualized by heatmap. (D–G) Western blot showed that HLJD administration reduced the protein expression of peptidylarginine deiminase 4 (PAD4) (E), p‐P38/P38 (F), p‐ extracellular signal‐regulated kinase (ERK)/ERK (G). *n* = 3 for immunofluorescence and western blot, *n* = 6 for transcriptomics.

We commenced by utilizing immunofluorescence to assess the expression of MPO^+^CitH3^+^ positive area, thereby gauging the impact of HLJD intervention on NETs in the brain tissue of MCAO/R model rats. The results illustrated that, in comparison to the Sham group, the MCAO/R group exhibited a significant increase in the expression of MPO^+^CitH3^+^ positive area, signifying an exacerbation in NETs deposition within the infarction area. However, following HLJD intervention, the expression of MPO^+^CitH3^+^ positive area significantly diminished **(**Figure 4A,B **)**.

**FIGURE 5:**
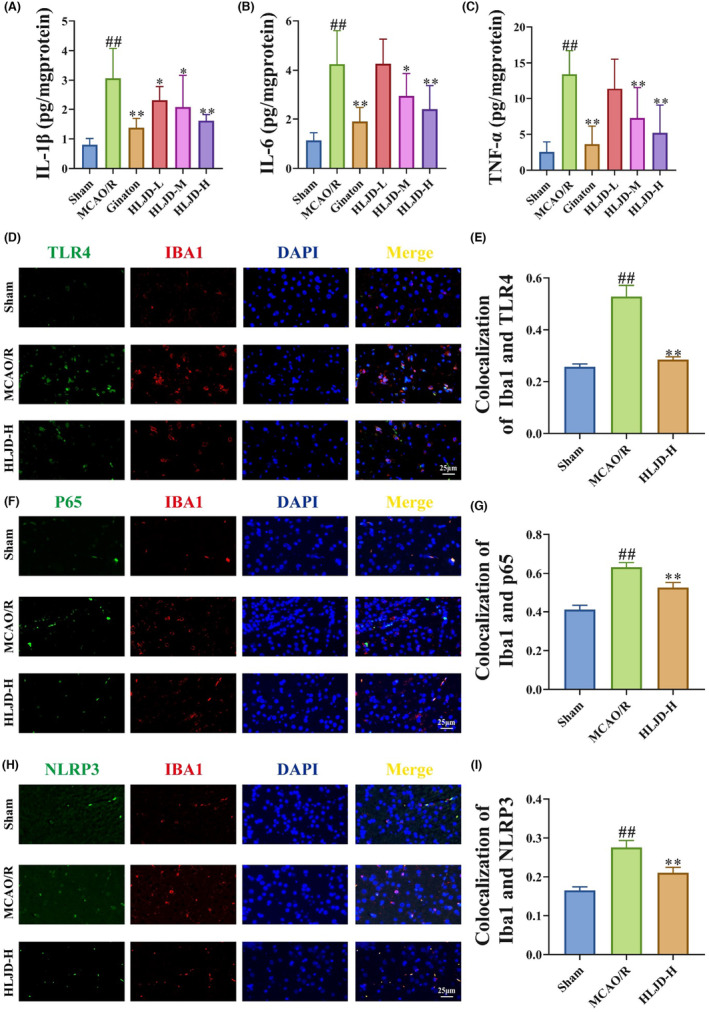
Huanglian Jiedu decoction (HLJD) treatment inhibited neuroinflammation in MCAO/R rats. (A–C) Enzyme‐linked immunosorbent assay (ELISA) test showed that HLJD intervention decreased the levels of interleukin (IL)‐1β (a), IL‐6 (B) and TNF‐α (C) in brain tissue homogenate. (D–I) Immunofluorescence results showed that HLJD treatment decreased positive expression areas of Iba1^+^ toll‐like receptor 4 (TLR4)^+^ (D, E), Iba1^+^p65^+^ (F, G) and Iba1^+^ NOD‐like receptor thermal protein domain associated protein 3 (NLRP3)^+^ (H, I) (Magnification: 400×). *n* = 9 for ELISA and *n* = 3 for immunofluorescence.

Subsequently, we delved into the transcriptome data to analyse the expression of NET formation‐related genes, including *Ncf4*, *Ncf2, Itgam*, *C5ar1*, *Casp1*, *Fcgr1a*, *Fcgr2a*, *Tlr8*, *Itgb3*, *Tlr2* and *Tlr4*. These genes were significantly up‐regulated in the MCAO/R group but displayed significant down‐regulation after HLJD intervention **(**Figure 4C **)**. Notably, we also identified PAD4, P38 and ERK as essential downstream molecules in the NET formation process. These molecules have been previously established to play pivotal roles in NET formation.^49^ To further elucidate their status, we employed western blot to examine the expression levels of PAD4, phosphorylated P38 and phosphorylate ERK in brain tissue. Our results revealed that, in contrast to the Sham group, the MCAO/R group displayed significant increases in the levels of PAD4, p‐P38/P38 and p‐ERK/ERK, which were all significantly reduced following HLJD intervention **(**Figure 4D–4G **)**. Importantly, PAD4, P38 and ERK are known not only to promote NET formation but also to activate microglia cells^18^ and the TLR4,^50^ NF‐κB,^51^ and NLRP3 signalling pathways,^52^ thereby promoting the development of inflammation. Therefore, we proceeded to assess the levels of inflammatory factors in brain tissue and employed immunofluorescence staining to investigate TLR4, p65, and NLRP3 levels in microglia cells. Our findings demonstrate that the levels of inflammatory factors (IL‐1β, IL‐6 and TNF‐α) in the brain tissue of MCAO/R rats were significantly higher than those in the Sham group, yet HLJD intervention led to a significant reduction in these inflammatory factor levels (Figure 5A–C). Furthermore, in comparison to the Sham group, the MCAO/R group exhibited a significant increase in the expression of Iba1^+^TLR4^+^, Iba1^+^p65^+^ and Iba1^+^NLRP3^+^ positive area, while HLJD intervention effectively reversed this outcome (Figure 5D–I).

### 3.4 HLJD activated the GABAergic synapses, improved nerve cell damage and fostered nerve cell proliferation

**FIGURE 6:**
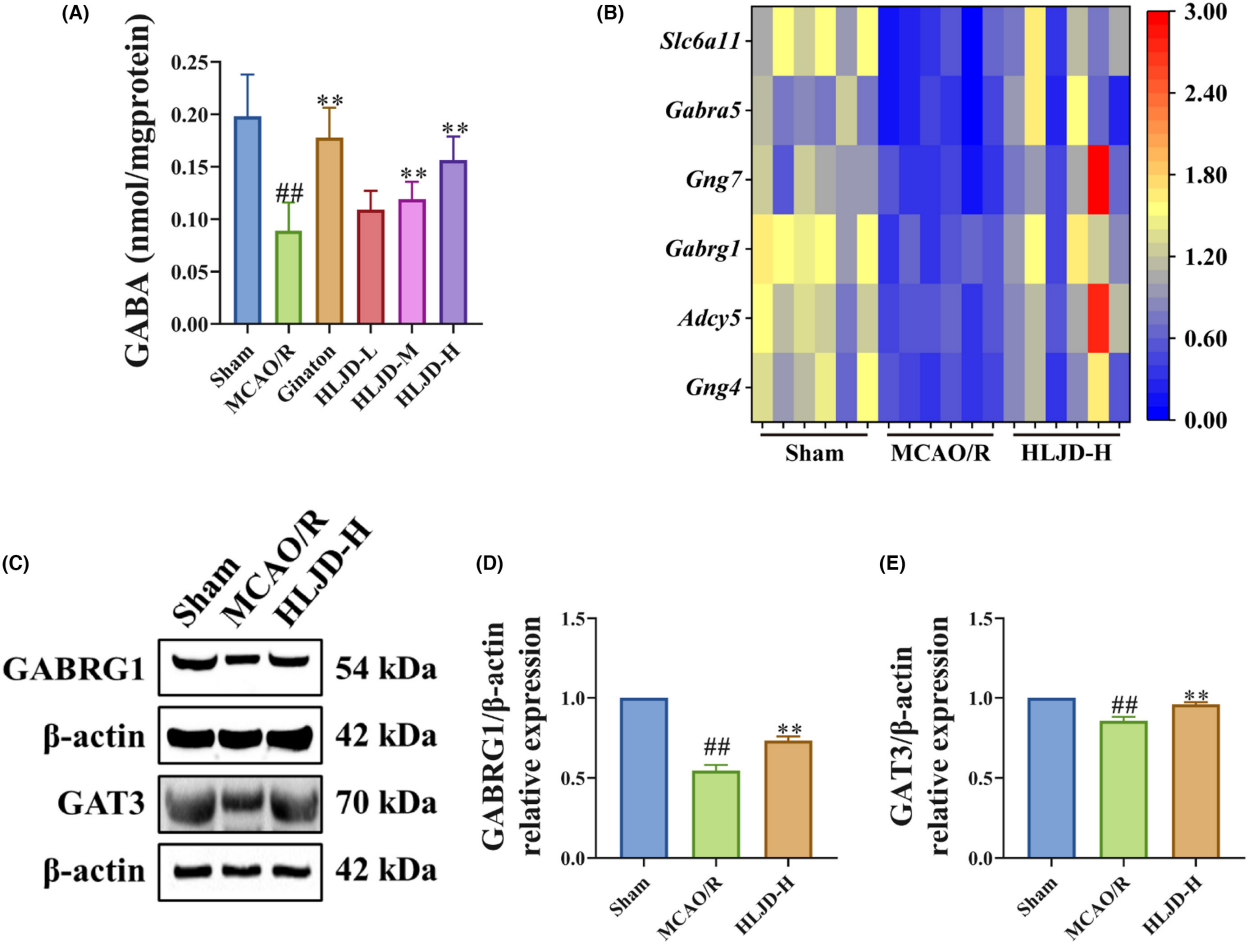
Huanglian Jiedu decoction (HLJD) treatment activated the GABAergic synapse in MCAO/R rats. (A) The ELISA test showed that HLJD intervention increased the level of gamma‐aminobutyric acid (GABA). (B) Differentially expressed genes involving in GABAergic synapse obtained from transcriptomics were visualized by heatmap. (C–E) Western blot showed that HLJD administration increased the protein expression of gamma‐amino butyric acid type A receptor gamma1 subunit (GABRG1) (E) and GABA transporter 3 (GAT3) (f). *n* = 6 for transcriptomics and *n* = 3 for western blot.

**FIGURE 7:**
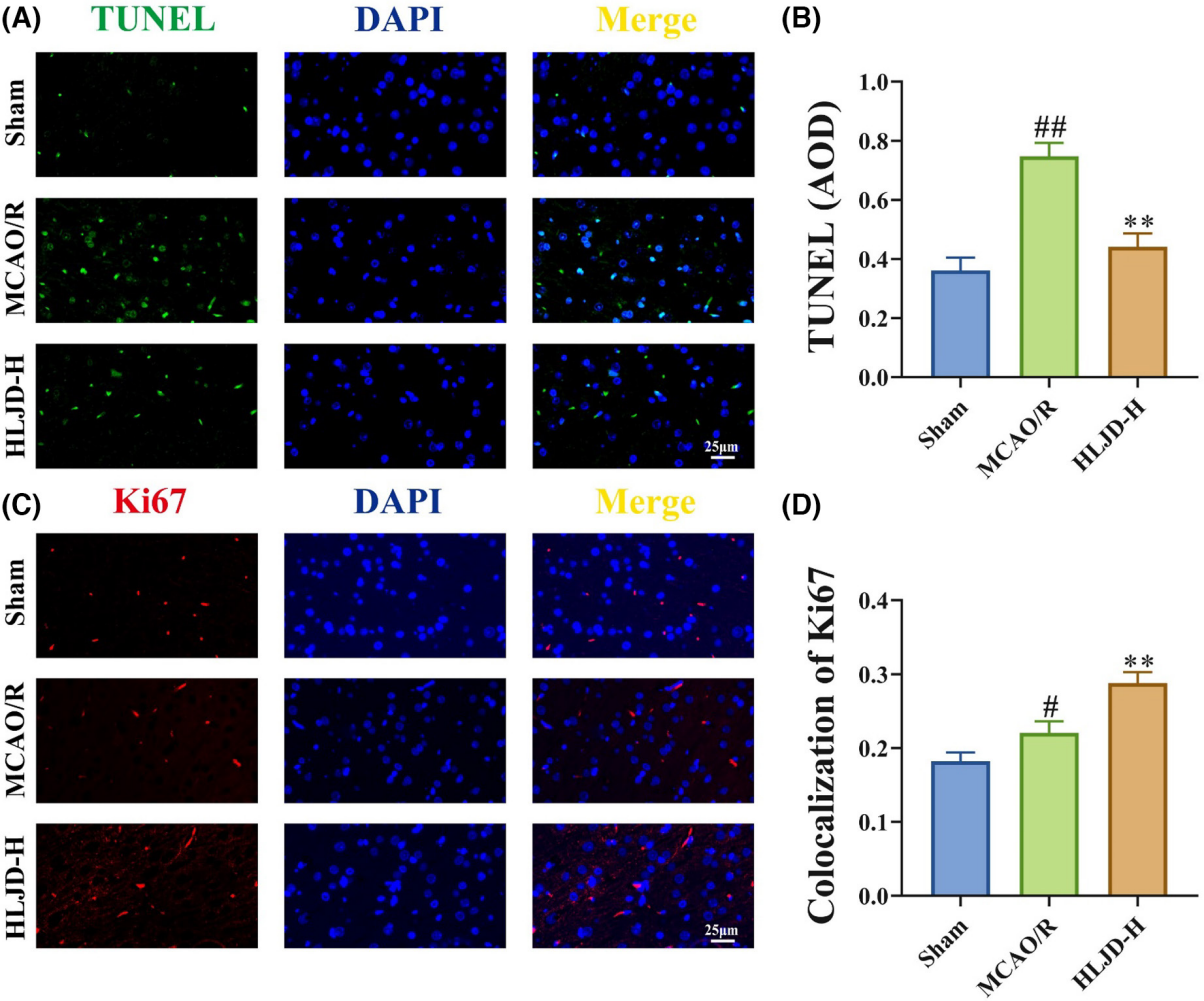
Huanglian Jiedu decoction (HLJD) treatment improved nerve cell damage and fostered nerve cell proliferation in MCAO/R rats. (A, B) Terminal deoxynucleotidyl transferase (TdT) dUTP Nick‐End Labelling (TUNEL) staining showed that HLJD intervention ameliorated apoptosis in brain. (C, D) Immunofluorescence of Ki67 indicated that HLJD intervention promoted cell proliferation in brain. Magnification: 400×, *n* = 3 per group.

We first assessed the GABA level in the brain tissue of each group, revealing that the GABA level in MCAO/R rats decreased compared to the Sham group. However, HLJD intervention significantly increased GABA levels **(**Figure 6A **)**. Furthermore, transcriptomic analysis uncovered a significant down‐regulation of genes related to GABAergic synapse, including *Gng4*, *Adcy5*, *Gabrg1*, *Gng7*, *Gabra5* and *Slc6a11* in MCAO/R rats, with HLJD intervention leading to a significant up‐regulation of these genes **(**Figure 6B **)**. The distribution and interaction of these genes within the GABAergic synapse network suggested a pivotal role for GABRG1 in the activation and development of GABAergic synapse, while SLC6A11 (also known as GAT3) emerged as a crucial GABA transporter.^53^ To corroborate these findings, we employed western blot to examine the levels of GABRG1 and GAT3 in the brain tissue. Our results indicated a reduction in the levels of GABRG1 and GAT3 in the MCAO/R group compared to the Sham group, while HLJD intervention increased their expression **(**Figure 6C–6E **)**. Additionally, previous studies have demonstrated that GABA plays a role in mediating neural cell injury and proliferation.^48^ Therefore, we conducted TUNEL staining and Ki67 staining to evaluate cell injury and proliferation in brain. The TUNEL staining revealed a significantly higher positive region in MCAO/R rats in comparison to the Sham group, with HLJD intervention resulting in a reduction in the positive region **(**Figure 7A, B **)**. The Ki67 staining, on the other hand, demonstrated an increase in the Ki67 positive area in brain tissue following HLJD intervention (Figure 7C,D).

## 4 DISCUSSION

Ischemic stroke, predominantly triggered by vascular embolism and compromised blood flow, culminates in cerebral ischemia, hypoxia and consequential brain injury. Numerous studies have documented the substantial efficacy of HLJD in treating ischemic stroke,^31^, ^32^, ^33^, ^34^ yet its anti‐inflammatory effects and mechanisms in the context of ischemic stroke remain unexplored. The present study was undertaken to investigate the impact of HLJD on ischemia‐induced cerebral injury in an MCAO/R rat model. Bederson's scale score,^36^, ^37^ postural reflex test,^38^ and asymmetry score^39^ are conventional tools for assessing neural function damage and recovery in rats. Our results suggest that HLJD promotes the recovery of neural function in MCAO/R rats. Furthermore, ischemia and hypoxia result in a significant increase in brain infarction area and severe neuronal damage in the cerebral cortex, characterized by disordered neuronal arrangement, nuclear condensation and the disappearance of Nissl bodies.^54^ Our findings indicate that HLJD alleviates these symptoms and fosters recovery from ischemia‐induced cerebral injury in MCAO/R rats. Notably, we employed Ginaton as a positive control^32^ and observed that high‐dose HLJD exhibited a similar effect to Ginaton in treating ischemic stroke.

Moreover, our transcriptomic analysis allowed us to delve deeper into the mechanisms underlying high‐dose HLJD's efficacy in improving ischemia‐induced cerebral injury. Building upon the KEGG pathway enrichment analysis of transcriptomics data, down‐regulated genes following HLJD treatment could be classified into inflammation, injury and repair, coagulation disorders and endothelial dysfunction processes. Interestingly, NETs formation has been shown with strong correlations with these pathological processes.^18^, ^45^, ^46^, ^47^ In addition, up‐regulated genes following HLJD treatment were mainly associated with pathways involving in neural transmission. The GABAergic synapse has been demonstrated to be crucial in regulating neural transmission.^48^ Therefore, we proceeded to validate the effects of HLJD on NETs formation and GABAergic synapse. NETs have been recognized for their unique role in the development of ischemic stroke.^18^, ^55^, ^56^ These extracellular fibrous mesh structures, composed of DNA, histones and neutrophil granule proteins, serve the function of trapping and killing pathogens.^56^ Research has indicated that NETs can exacerbate ischemic neural damage and serve as an early diagnostic marker for ischemic stroke. Patients with ischemic stroke have been shown to exhibit significantly elevated levels of NETs compared to healthy individual.^55^, ^57^, ^58^ CitH3 and extracellular DNA serve as markers of NETs formation, detectable through co‐expression of CitH3 and the neutrophil marker MPO.^49^ Our results demonstrate that HLJD intervention effectively reduces NETs deposition. Furthermore, HLJD intervention down‐regulated the expression of NETs‐related genes, including *Ncf4*, *Ncf2, Itgam*, *C5ar1*, *Casp1*, *Fcgr1a*, *Fcgr2a*, *Tlr8*, *Itgb3*, *Tlr2* and *Tlr4*. Among these genes, *Ncf2*, *Ncf4*, *Fcgr1a*, *Fcgr2a*, *C5ar1*, *Itgb3* and *Itgam* can promote PAD4 expression through different pathways, while *Tlr2* and *Tlr4* can promote P38 and ERK expression. *Fcgr1a* and *Fcgr2a* can further promote ERK expression. Therefore, we conducted a more in‐depth analysis of PAD4, P38 and ERK. PAD4 is an essential enzyme in NETs formation, mediating histone citrullination and chromatin decondensation.^59^ Excessive PAD4 expression in the cerebral cortex during ischemic stroke has been linked to increased NETs, accompanied by decreased neovascularization and enhanced blood–brain barrier damage. Inhibiting PAD4 activity through medication can deplete neutrophils, fostering vascular repair, enhancing neovascularization and promoting neural function recovery.^56^ Previous research has highlighted that phosphorylation activation of P38 and ERK can stimulate the activation of neutrophil membrane‐related oxidases, thereby promoting MPO production and PAD4 activation. Elevated PAD4 activation ultimately fuels NETs formation.^49^, ^60^ Intriguingly, our findings reveal that HLJD intervention significantly reduces the expression of PAD4, along with phosphorylated P38 and ERK, implying that HLJD effectively curtails NET formation and promotes neural cell repair post‐ischemia. Additionally, studies have elucidated that PAD4, P38 and ERK can activate various inflammatory pathways, including microglia, TLR4, NF‐κB and NLRP3 pathways, culminating in an inflammatory cytokine storm that exacerbates ischemia‐induced cerebral injury.^18^, ^50^, ^51^, ^52^ Our results indicate that HLJD intervention effectively curtails inflammatory factor levels and the expression of related inflammatory pathways. This suggests that HLJD intervention may alleviate ischemia‐induced cerebral injury by inhibiting NETs formation and related inflammatory pathways. Furthermore, geniposide, a major component in HLJD, has been demonstrated to inhibit NET formation in acute kidney injury model.^61^ Berberine has been shown to suppress neuroinflammation through inhibiting microglia activation.^62^ Whether these components exhibit NET and microglia inhibitory effects on MCAO/R model still needs to be further studied.

Additionally, our study revealed a significant up‐regulation in the GABA level within the brain tissue of MCAO/R rats following HLJD intervention, consistent with prior research findings.^63^ Extensive research has underscored the pivotal role of GABA in the recovery from ischemia‐induced cerebral injury.^64^, ^65^ GABA is recognized as an inhibitory neurotransmitter in the post‐ischemic brain, effectively tempering neural excitation and mitigating neural cell damage caused by excitatory amino acids.^64^ Furthermore, our analysis identified a pronounced up‐regulation of GABA pathway‐related genes, including *Gng4*, *Adcy5*, *Gabrg1*, *Gng7*, *Gabra5* and *Slc6a11*. These genes are instrumental in maintaining the dynamic balance of GABA within the body. Notably, *Gabrg1* and *Slc6a11* govern GABA receptors and transporters, which act as vital components in the GABA‐modulated neural system.^64^, ^66^ Consequently, we conducted a comprehensive examination of the GABA receptor, GABRG1 and the GABA transporter, SLC6A11. GABRG1, a member of the ligand‐gated receptor family, mediates signal transduction by directly opening ion channels for transmembrane transmission without the need for second messenger systems. It primarily facilitates swift inhibitory synaptic transmission.^66^ When GABRG1 function is compromised, it can lead to heightened neural excitation, potentially resulting in post‐stroke spasticity.^64^ On the other hand, SLC6A11, also known as GAT3, fulfils a unique role in GABA transport. It predominantly resides in the synaptic and perisynaptic membranes of neurons and glial cells, where it transports GABA into the cytoplasm to enhance its neural inhibitory effect.^53^ Our study establishes that HLJD intervention effectively elevates the expression of GABRG1 and GAT3, thereby enhancing the inhibitory synaptic transmission function of GABA and ameliorating neural excitation. Furthermore, previous research has illuminated the neuroprotective properties of GABA, which inhibits neural cell damage and stimulates proliferation.^64^ Notably, our findings reveal that HLJD intervention effectively mitigates neural cell damage and promotes neural cell proliferation. This implies that HLJD intervention holds the potential to foster the repair of ischemia‐induced cerebral injury by facilitating GABA secretion and transmission.

## 5 CONCLUSION

**FIGURE 8:**
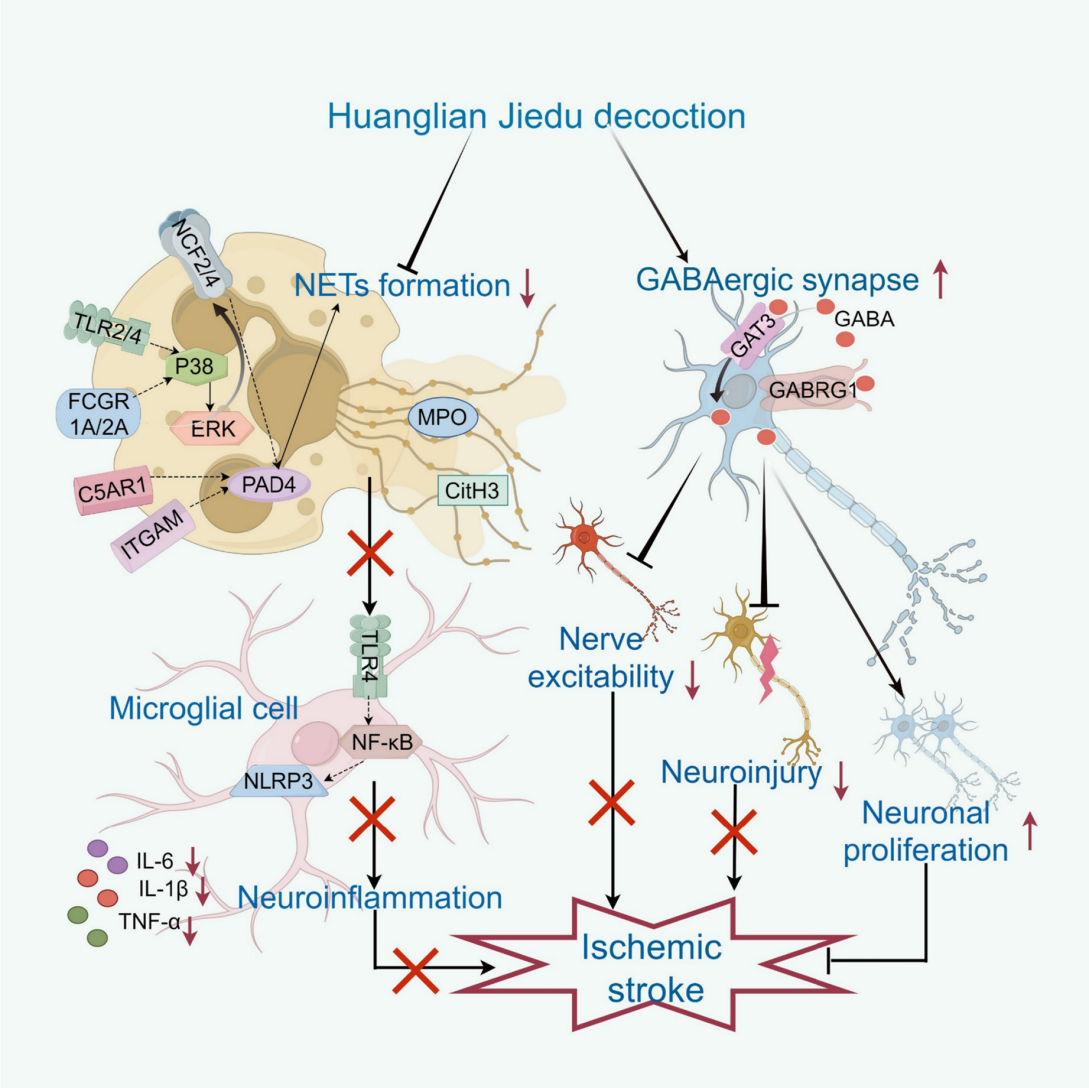
Huanglian Jiedu decoction intervention effectively inhibits neuroinflammation by mitigating NET formation, and concurrently inhibits nerve cell damage and fosters nerve cell proliferation through activating GABAergic synapses, and finally alleviates ischemia‐induced cerebral injury.

In conclusion, our study demonstrates that HLJD intervention exerts a multifaceted positive impact on ischemia‐induced cerebral injury in MCAO/R rats. This intervention effectively inhibits neuroinflammation by mitigating NET formation, and concurrently improves nerve cell damage and fosters nerve cell proliferation through activating GABAergic synapses (Figure 8). These findings collectively underline the potential therapeutic value of HLJD in the context of ischemic stroke.

There are also some limitations in our study. Our results revealed several mechanisms of HLJD on MCAO/R rats. However, as HLJD contains several components, the detailed mechanisms of active components in HLJD on NET formation and GABAergic synapses still require to be studied. Further in vitro models such as NETs induction, oxygen glucose deprivation/re‐oxygenation induced neuron injury models can be used to further elucidate the in‐depth mechanism of active components in HLJD. Furthermore, novel techniques such as single‐cell sequencing, spatial transcriptomics can also be useful to explain the mechanism of HLJD on ischemic stroke.

## AUTHOR CONTRIBUTIONS

**Youxiang Cui:** Writing – original draft (equal). **Mingyue Cui:** Writing – review and editing (equal). **Leilei Wang:** Formal analysis (equal). **Ning Wang:** Supervision (equal). **Yao Chen:** Resources (equal). **Shuquan Lv:** Investigation (equal). **Limin Zhang:** Funding acquisition (equal). **Congai Chen:** Validation (equal). **Yanwen Yang:** Software (equal). **Feng Wang:** Methodology (equal). **Lichun Wang:** Visualization (equal). **Huantian Cui:** Conceptualization (equal); funding acquisition (equal); resources (equal); supervision (equal).

## FUNDING INFORMATION

This work was supported by the National Natural Science Foundation of China (U21A20400).

## CONFLICT OF INTEREST STATEMENT

All authors declare no conflict of interests.

## Supporting information


Data S1.

## Data Availability

Data will be made available on request.
