# Supplementary material for: Huanglian Jiedu decoction alleviates ischemia‐induced cerebral injury in rats by mitigating NET formation and activiting GABAergic synapses
Source: J Cell Mol Med. 2024 Aug 4;28(15):e18528. doi: 10.1111/jcmm.18528 (PMC11298410; doi:10.1111/jcmm.18528)
Supplement: Supplementary file 1 — Data S1. [file JCMM-28-e18528-s001.docx]

**1 Reagents**

Ginaton (H20090365) were purchased from Dr. Willmar Schwabe GmbH & Co. KG (Karlsruhe, Germany). ELISA kits for IL-1β (ml037361), IL-6 (ml102828), TNF-α (ml002859), and GABA (ml092747) were purchased from Shanghai Enzyme-linked Biotechnology Co., Ltd. (Shanghai, China). Immunofluorescence primary antibodies: mouse anti-MPO (ab90810), rabbit anti-CitH3 (ab219407), rabbit anti-Iba1 (ab178847) and mouse anti-TLR4 (ab22048), rabbit anti-Ki67(ab16667), mouse anti-Iba1 (ab283319) were purchased from Abcam (Shanghai, China); mouse anti-p65 (6956) was purchased from Cell Signaling Technology (Shanghai, China); rabbit anti-NLRP3 (K010074P) was purchased from Solarbio (Beijing, China). Western blot primary antibodies: PAD4 (ab214810) was purchased from Abcam (Shanghai, China), p-P38 (9211), P38 (9212), p-ERK (9101), ERK (9102), β-actin (8457) were purchased from Cell Signaling Technology (Shanghai, China), GABRG1 (110812) and GAT3 (25438) were purchased from NovoPro (Shanghai, China). Secondary antibody for goat anti-mouse IgG H&L (ab6785) and goat anti-rabbit IgG H&L (ab207995) were purchased from Abcam (Shanghai, China). Geniposide (HY-N0009), berberine (HY-18258), baicalin (HY-N0197) were purchased from MedChemExpress (Shanghai, China).

**2 Component analysis of HLJD by HPLC**

**2.1 Standard solution preparation**

The standards of berberine, baicalin, and geniposide were precisely weighed and dissolved in methanol to prepare the corresponding component standard storage solution with mass concentration of 0.108, 0.313, and 0.121 mg/mL. The standard solution can be obtained by taking an appropriate amount of the standard storage solution and mixing it. 1mL of HLJD was diluted to 5mL with 30% methanol, shaken and filtered with a 0.45μm filter. The filtrate was the HLJD assay solution.

**2.2 HPLC analytical method**

The chemical composition of the standard solution and HLJD was determined by High Performance Liquid Chromatography (HPLC) detector (Waters 2695, Waters, Shanghai, China), respectively. Chromatographic conditions include: Waters Symmetry Shield C18 chromatographic column (250 mm × 4.6 mm, 5 µm), mobile phase consisted of acetonitrile and 0.2% phosphoric acid solution. The gradient elution program: 0-10 min, 10-22% acetonitrile; 10-40 min, 22-23% acetonitrile; 40-45 min, 23-24% acetonitrile; 45-60 min, 24% acetonitrile. The flow rate was 1 mL/min, the detection wavelength was 260 nm, the column temperature was 35 °C, and the injection volume was 10 µL. HPLC analysis of standards as well as HLJD was shown in **Figure S1**.

**Figure S1 HPLC analysis of standards and HLJD.**

**3 Neurological function evaluation**

**Table S1 Neurological function evaluation**

| Score | Bederson's scale score | Postural reflex test | Asymmetry score |
| --- | --- | --- | --- |
| 0 | No neurological functional impairment | Right forelimb showed ventral and lateral stretch | The front limb of rats with an unconstrained hanging freely brushed the edge of a table for 20 times.  $Asymmetry score=\frac{Left-Right}{Both+Left+Right}\times100\%$ |
| 1 | Continuous torso flexion to the right; | Right forelimb extended at an angle |  |
| 2 | Weakened resistance to contralateral push without turning | Right forelimb affixed to the chest with twisted body |  |
| 3 | Spontaneous circling around affected limbs under the condition of free activity | / |  |
| 4 | No spontaneous motor activity and occurring flaccid paralysis | / |  |

**4** **Differentially expressed genes in brain following HLJD treatment**

**Table S2 HLJD intervention downregulated 263 genes**

| Gene number | Gene name | log2FoldChange | | padj | |
| --- | --- | --- | --- | --- | --- |
|  |  | MCAO/R vs Sham | HLJD-H vs MCAO/R | MCAO/R vs Sham | HLJD-H vs MCAO/R |
| 1 | Ly86 | 2.370 | -1.744 | 0.000 | 0.032 |
| 2 | Sapcd1 | 1.508 | -1.179 | 0.000 | 0.045 |
| 3 | Medag | 2.155 | -2.143 | 0.000 | 0.028 |
| 4 | Cstb | 1.511 | -1.289 | 0.000 | 0.028 |
| 5 | Orai1 | 1.144 | -1.076 | 0.000 | 0.039 |
| 6 | Adamts1 | 3.444 | -2.043 | 0.000 | 0.038 |
| 7 | Nrros | 1.671 | -1.298 | 0.000 | 0.047 |
| 8 | Arntl2 | 3.225 | -2.809 | 0.000 | 0.012 |
| 9 | Mcm4 | 1.440 | -1.053 | 0.000 | 0.039 |
| 10 | Plac8 | 4.159 | -2.150 | 0.000 | 0.038 |
| 11 | Serpinb8 | 6.374 | -3.553 | 0.000 | 0.023 |
| 12 | Tmem100 | 2.330 | -1.429 | 0.000 | 0.044 |
| 13 | Litaf | 2.019 | -1.496 | 0.000 | 0.046 |
| 14 | Ptgs2 | 1.792 | -1.793 | 0.003 | 0.045 |
| 15 | Pla2g4a | 2.198 | -1.465 | 0.000 | 0.049 |
| 16 | Cxcl2 | 4.744 | -5.023 | 0.005 | 0.029 |
| 17 | Cxcl1 | 6.262 | -3.142 | 0.000 | 0.039 |
| 18 | Mnda | 5.709 | -3.701 | 0.000 | 0.025 |
| 19 | Tnfrsf12a | 3.023 | -2.336 | 0.000 | 0.026 |
| 20 | Srpx2 | 1.576 | -1.567 | 0.000 | 0.021 |
| 21 | Gpr65 | 3.782 | -2.658 | 0.000 | 0.024 |
| 22 | Rgs1 | 4.703 | -2.751 | 0.000 | 0.029 |
| 23 | Il2rg | 2.514 | -2.046 | 0.000 | 0.046 |
| 24 | Pfn1 | 1.176 | -1.005 | 0.000 | 0.037 |
| 25 | Sh3pxd2b | 1.588 | -1.519 | 0.000 | 0.039 |
| 26 | Trib1 | 1.822 | -1.588 | 0.000 | 0.028 |
| 27 | Ifitm1 | 2.677 | -2.241 | 0.000 | 0.014 |
| 28 | Ddx39a | 1.755 | -1.340 | 0.000 | 0.042 |
| 29 | Myc | 2.596 | -1.840 | 0.000 | 0.039 |
| 30 | Nxt1 | 1.497 | -1.163 | 0.000 | 0.042 |
| 31 | Upp1 | 3.083 | -2.096 | 0.000 | 0.011 |
| 32 | Adamts8 | 1.346 | -1.424 | 0.002 | 0.033 |
| 33 | Fam110c | 2.529 | -2.327 | 0.001 | 0.039 |
| 34 | Arhgdib | 2.310 | -1.633 | 0.000 | 0.044 |
| 35 | Il1rn | 3.969 | -2.991 | 0.000 | 0.012 |
| 36 | Rln3 | 2.755 | -3.167 | 0.008 | 0.033 |
| 37 | Cd44 | 3.856 | -2.308 | 0.000 | 0.039 |
| 38 | Ptges | 3.397 | -2.112 | 0.000 | 0.030 |
| 39 | Ccr1 | 4.917 | -3.920 | 0.000 | 0.009 |
| 40 | Cst7 | 5.991 | -3.963 | 0.000 | 0.025 |
| 41 | Ncf4 | 4.525 | -2.702 | 0.000 | 0.025 |
| 42 | Lif | 4.777 | -3.357 | 0.000 | 0.013 |
| 43 | Plin2 | 3.325 | -2.219 | 0.000 | 0.040 |
| 44 | Casp1 | 2.295 | -1.610 | 0.000 | 0.023 |
| 45 | Cd63 | 2.421 | -1.509 | 0.000 | 0.039 |
| 46 | Apold1 | 2.127 | -1.216 | 0.000 | 0.026 |
| 47 | Fos | 1.543 | -1.399 | 0.001 | 0.044 |
| 48 | Lyn | 2.122 | -1.431 | 0.000 | 0.039 |
| 49 | Tagln2 | 2.547 | -1.927 | 0.000 | 0.034 |
| 50 | Gprc5a | 3.724 | -2.466 | 0.000 | 0.016 |
| 51 | Rbms1 | 1.413 | -1.266 | 0.000 | 0.018 |
| 52 | Emp1 | 3.575 | -2.344 | 0.000 | 0.018 |
| 53 | Plbd1 | 3.676 | -2.395 | 0.000 | 0.043 |
| 54 | Tlr2 | 1.937 | -1.706 | 0.000 | 0.046 |
| 55 | Tmem88 | 1.599 | -1.596 | 0.000 | 0.011 |
| 56 | Fgr | 4.005 | -2.367 | 0.000 | 0.046 |
| 57 | Pnp | 1.047 | -1.059 | 0.000 | 0.011 |
| 58 | Tlr4 | 1.991 | -1.522 | 0.000 | 0.048 |
| 59 | Sphk1 | 2.872 | -2.284 | 0.000 | 0.014 |
| 60 | Ccn5 | 2.004 | -1.786 | 0.001 | 0.040 |
| 61 | Dap | 1.293 | -1.020 | 0.000 | 0.039 |
| 62 | Ppp2r1b | 1.412 | -1.026 | 0.000 | 0.038 |
| 63 | Ccl3 | 5.294 | -3.662 | 0.000 | 0.046 |
| 64 | Ccl4 | 5.679 | -3.317 | 0.000 | 0.042 |
| 65 | Nkain1 | 1.609 | -1.200 | 0.000 | 0.049 |
| 66 | Fam180a | 1.309 | -1.625 | 0.029 | 0.043 |
| 67 | Fblim1 | 2.639 | -2.008 | 0.000 | 0.033 |
| 68 | Sox7 | 2.527 | -2.174 | 0.000 | 0.038 |
| 69 | Txn1 | 1.214 | -1.019 | 0.000 | 0.039 |
| 70 | Spi1 | 2.525 | -1.863 | 0.000 | 0.029 |
| 71 | Itgb7 | 5.135 | -3.435 | 0.000 | 0.030 |
| 72 | Tgfbi | 4.179 | -2.896 | 0.000 | 0.048 |
| 73 | Bid | 1.539 | -1.156 | 0.000 | 0.041 |
| 74 | Eif4ebp1 | 1.751 | -1.444 | 0.000 | 0.029 |
| 75 | Rhoc | 2.294 | -1.425 | 0.000 | 0.045 |
| 76 | Slc25a43 | 2.424 | -1.330 | 0.000 | 0.044 |
| 77 | Fgl2 | 3.757 | -2.029 | 0.000 | 0.036 |
| 78 | Tgm2 | 2.721 | -1.607 | 0.000 | 0.030 |
| 79 | Nr1h3 | 1.707 | -1.404 | 0.000 | 0.028 |
| 80 | Kcnj8 | 1.542 | -1.086 | 0.000 | 0.021 |
| 81 | Dok3 | 3.281 | -1.864 | 0.000 | 0.034 |
| 82 | Kdelr3 | 1.494 | -1.069 | 0.000 | 0.047 |
| 83 | Slc31a2 | 2.135 | -1.491 | 0.000 | 0.030 |
| 84 | Pdlim7 | 1.116 | -1.146 | 0.000 | 0.030 |
| 85 | Enpp3 | 3.311 | -2.823 | 0.000 | 0.033 |
| 86 | Sbno2 | 2.609 | -1.778 | 0.000 | 0.036 |
| 87 | Dll4 | 1.202 | -1.312 | 0.002 | 0.033 |
| 88 | Hmox1 | 5.124 | -3.494 | 0.000 | 0.005 |
| 89 | Fn1 | 3.087 | -1.807 | 0.000 | 0.034 |
| 90 | Ptpn6 | 2.531 | -1.632 | 0.000 | 0.044 |
| 91 | Ccn1 | 4.188 | -2.684 | 0.000 | 0.039 |
| 92 | Gja4 | 1.780 | -1.488 | 0.000 | 0.043 |
| 93 | Il1r2 | 6.293 | -3.765 | 0.000 | 0.037 |
| 94 | Lox | 2.569 | -1.771 | 0.000 | 0.039 |
| 95 | Anpep | 2.882 | -2.226 | 0.000 | 0.035 |
| 96 | Arl11 | 3.397 | -2.323 | 0.000 | 0.039 |
| 97 | Mapkapk3 | 1.328 | -1.147 | 0.000 | 0.033 |
| 98 | Slc11a1 | 2.556 | -2.027 | 0.000 | 0.042 |
| 99 | Myl12a | 2.398 | -1.481 | 0.000 | 0.042 |
| 100 | Nabp1 | 1.186 | -1.186 | 0.007 | 0.042 |
| 101 | Lpar6 | 1.680 | -1.348 | 0.000 | 0.043 |
| 102 | Cd2 | 4.158 | -3.745 | 0.002 | 0.044 |
| 103 | Niban2 | 1.486 | -1.136 | 0.000 | 0.042 |
| 104 | Tgif1 | 2.425 | -1.624 | 0.000 | 0.050 |
| 105 | LOC108349548 | 1.380 | -1.083 | 0.000 | 0.044 |
| 106 | Slc1a5 | 1.690 | -1.563 | 0.000 | 0.033 |
| 107 | Mafb | 2.447 | -1.754 | 0.000 | 0.046 |
| 108 | Sox18 | 1.269 | -1.091 | 0.001 | 0.050 |
| 109 | Frrs1 | 2.109 | -1.713 | 0.000 | 0.036 |
| 110 | Vasp | 1.492 | -1.215 | 0.000 | 0.047 |
| 111 | Ctsc | 3.425 | -2.236 | 0.000 | 0.042 |
| 112 | Angptl2 | 2.128 | -1.932 | 0.000 | 0.017 |
| 113 | Angpt2 | 2.554 | -1.807 | 0.000 | 0.038 |
| 114 | Ptgir | 2.523 | -1.832 | 0.000 | 0.048 |
| 115 | Loxl2 | 1.256 | -1.274 | 0.002 | 0.031 |
| 116 | Pla2g2d | 2.093 | -2.152 | 0.000 | 0.033 |
| 117 | Serpinh1 | 1.234 | -1.118 | 0.000 | 0.033 |
| 118 | Igsf6 | 3.863 | -2.589 | 0.000 | 0.036 |
| 119 | Il11 | 4.954 | -2.886 | 0.000 | 0.046 |
| 120 | Gja5 | 3.810 | -2.526 | 0.000 | 0.039 |
| 121 | Rsu1 | 1.258 | -1.119 | 0.000 | 0.010 |
| 122 | Dennd2d | 2.472 | -2.387 | 0.006 | 0.034 |
| 123 | Cd53 | 2.572 | -1.926 | 0.000 | 0.039 |
| 124 | Adam8 | 3.614 | -3.050 | 0.000 | 0.026 |
| 125 | F2rl1 | 2.427 | -2.199 | 0.000 | 0.014 |
| 126 | Vwa1 | 2.018 | -1.208 | 0.000 | 0.042 |
| 127 | Tubb6 | 4.311 | -2.710 | 0.000 | 0.017 |
| 128 | Ctsl | 1.728 | -1.184 | 0.000 | 0.048 |
| 129 | Cdc42ep5 | 1.242 | -1.123 | 0.000 | 0.000 |
| 130 | Hbegf | 3.074 | -2.248 | 0.000 | 0.025 |
| 131 | Ch25h | 2.448 | -1.669 | 0.000 | 0.044 |
| 132 | Pvr | 2.327 | -1.881 | 0.000 | 0.026 |
| 133 | Nupr1 | 4.535 | -4.030 | 0.000 | 0.011 |
| 134 | Ltbr | 1.419 | -1.124 | 0.000 | 0.045 |
| 135 | P2ry6 | 2.500 | -1.868 | 0.000 | 0.019 |
| 136 | Procr | 2.436 | -1.783 | 0.000 | 0.030 |
| 137 | Tax1bp3 | 1.913 | -1.261 | 0.000 | 0.049 |
| 138 | Scnn1a | 2.707 | -1.569 | 0.000 | 0.028 |
| 139 | Kcnn4 | 4.950 | -2.782 | 0.000 | 0.039 |
| 140 | AABR07068316.1 | 1.278 | -1.303 | 0.002 | 0.044 |
| 141 | Itgam | 2.838 | -1.796 | 0.000 | 0.044 |
| 142 | Stat3 | 1.720 | -1.131 | 0.000 | 0.044 |
| 143 | Gadd45b | 2.141 | -1.396 | 0.000 | 0.044 |
| 144 | Napsa | 3.907 | -2.250 | 0.000 | 0.049 |
| 145 | Folr2 | 3.911 | -2.437 | 0.000 | 0.030 |
| 146 | Bag3 | 2.033 | -1.080 | 0.000 | 0.044 |
| 147 | Lsp1 | 4.519 | -2.434 | 0.000 | 0.046 |
| 148 | Lgals7 | 1.896 | -1.669 | 0.000 | 0.030 |
| 149 | Adcy4 | 1.984 | -1.416 | 0.000 | 0.043 |
| 150 | Tgfb1 | 2.014 | -1.431 | 0.000 | 0.044 |
| 151 | Nme6 | 1.252 | -1.407 | 0.000 | 0.010 |
| 152 | Pyy | 4.505 | -4.504 | 0.002 | 0.032 |
| 153 | Ms4a6a | 4.049 | -2.187 | 0.000 | 0.026 |
| 154 | Fxyd5 | 2.672 | -1.928 | 0.000 | 0.025 |
| 155 | Emp3 | 3.363 | -2.221 | 0.000 | 0.026 |
| 156 | Fcgr1a | 3.122 | -2.258 | 0.000 | 0.028 |
| 157 | Rhoj | 1.667 | -1.240 | 0.000 | 0.047 |
| 158 | Cxcl11 | 2.754 | -2.820 | 0.001 | 0.016 |
| 159 | Hspb8 | 1.640 | -1.077 | 0.000 | 0.030 |
| 160 | AABR07066818.1 | 1.223 | -1.058 | 0.003 | 0.023 |
| 161 | S100a10 | 4.113 | -2.177 | 0.000 | 0.044 |
| 162 | Ybx1 | 1.527 | -1.167 | 0.000 | 0.041 |
| 163 | Fcer1g | 2.398 | -1.776 | 0.000 | 0.029 |
| 164 | Osm | 3.286 | -2.699 | 0.001 | 0.048 |
| 165 | Peg12 | 2.747 | -2.948 | 0.005 | 0.030 |
| 166 | Hopx | 1.575 | -1.139 | 0.000 | 0.042 |
| 167 | Ier5l | 1.256 | -1.011 | 0.000 | 0.016 |
| 168 | Stat6 | 1.450 | -1.190 | 0.000 | 0.049 |
| 169 | Prss35 | 1.802 | -1.150 | 0.000 | 0.039 |
| 170 | Clec5a | 3.780 | -3.955 | 0.000 | 0.000 |
| 171 | Glipr1 | 1.802 | -1.536 | 0.000 | 0.038 |
| 172 | Cxcl16 | 2.964 | -2.075 | 0.000 | 0.039 |
| 173 | Jaml | 1.108 | -1.235 | 0.006 | 0.042 |
| 174 | RGD1359290 | 1.205 | -1.176 | 0.043 | 0.004 |
| 175 | AABR07021402.1 | 3.190 | -2.159 | 0.000 | 0.044 |
| 176 | Cxcl3 | 7.550 | -6.582 | 0.008 | 0.028 |
| 177 | Slc28a2 | 2.839 | -2.299 | 0.000 | 0.023 |
| 178 | LOC100362366 | 1.109 | -1.304 | 0.006 | 0.044 |
| 179 | Gsap | 3.209 | -2.018 | 0.000 | 0.033 |
| 180 | LOC102555453 | 1.130 | -1.263 | 0.000 | 0.011 |
| 181 | LOC102555453 | 1.032 | -1.329 | 0.013 | 0.032 |
| 182 | Cish | 2.382 | -1.632 | 0.000 | 0.039 |
| 183 | Ccl12 | 5.625 | -4.008 | 0.001 | 0.046 |
| 184 | Clec4a2 | 6.433 | -4.362 | 0.000 | 0.023 |
| 185 | Ccl6 | 3.732 | -2.360 | 0.000 | 0.046 |
| 186 | LOC100359600 | 1.361 | -1.372 | 0.007 | 0.044 |
| 187 | LOC100362684 | 1.995 | -1.181 | 0.000 | 0.033 |
| 188 | LOC689899 | 1.354 | -1.118 | 0.003 | 0.038 |
| 189 | Slc25a45 | 1.717 | -1.408 | 0.000 | 0.005 |
| 190 | Sytl2 | 3.691 | -3.312 | 0.000 | 0.042 |
| 191 | Lexm | 2.140 | -2.596 | 0.027 | 0.031 |
| 192 | Irgm | 1.018 | -1.007 | 0.001 | 0.030 |
| 193 | LOC108349606 | 1.222 | -1.021 | 0.000 | 0.025 |
| 194 | F7 | 5.018 | -4.530 | 0.005 | 0.044 |
| 195 | Esam | 1.382 | -1.082 | 0.000 | 0.050 |
| 196 | Oas1f | 1.094 | -1.020 | 0.001 | 0.044 |
| 197 | Rasgrp4 | 2.037 | -2.358 | 0.000 | 0.014 |
| 198 | AABR07007121.1 | 1.097 | -1.161 | 0.028 | 0.039 |
| 199 | AC128792.1 | 1.757 | -1.657 | 0.003 | 0.048 |
| 200 | Slc16a3 | 2.883 | -2.074 | 0.000 | 0.025 |
| 201 | Cd68 | 3.973 | -2.832 | 0.000 | 0.030 |
| 202 | Vsig4 | 6.964 | -5.167 | 0.000 | 0.022 |
| 203 | Tlr1 | 4.083 | -2.332 | 0.000 | 0.019 |
| 204 | Cd86 | 2.883 | -1.886 | 0.000 | 0.034 |
| 205 | Dram1 | 2.081 | -1.454 | 0.000 | 0.022 |
| 206 | Cd200r1 | 2.513 | -1.877 | 0.000 | 0.048 |
| 207 | Slc37a2 | 1.990 | -1.713 | 0.000 | 0.044 |
| 208 | Plp2 | 4.027 | -2.280 | 0.000 | 0.048 |
| 209 | Ecscr | 3.343 | -2.573 | 0.000 | 0.029 |
| 210 | Mir675 | 8.464 | -3.270 | 0.000 | 0.043 |
| 211 | Clec4a1 | 3.487 | -2.196 | 0.000 | 0.037 |
| 212 | RGD1307182 | 3.314 | -3.164 | 0.005 | 0.049 |
| 213 | Milr1 | 3.120 | -3.206 | 0.000 | 0.025 |
| 214 | Cd300le | 5.041 | -3.945 | 0.000 | 0.016 |
| 215 | Cnn2 | 3.233 | -1.892 | 0.000 | 0.043 |
| 216 | Thbs1 | 3.343 | -3.165 | 0.004 | 0.047 |
| 217 | Tlr8 | 3.632 | -2.629 | 0.000 | 0.046 |
| 218 | Icos | 1.587 | -2.205 | 0.023 | 0.039 |
| 219 | Cd300c2 | 8.203 | -6.545 | 0.000 | 0.010 |
| 220 | Cacna1s | 2.830 | -2.177 | 0.000 | 0.030 |
| 221 | Fcgr2a | 3.111 | -2.344 | 0.000 | 0.007 |
| 222 | PCOLCE2 | 2.206 | -1.872 | 0.000 | 0.014 |
| 223 | Bdkrb2 | 2.191 | -1.713 | 0.006 | 0.048 |
| 224 | AABR07006269.1 | 4.528 | -2.235 | 0.000 | 0.028 |
| 225 | LOC103690108 | 23.051 | -22.297 | 0.000 | 0.000 |
| 226 | C5ar1 | 4.249 | -2.843 | 0.000 | 0.016 |
| 227 | AC128859.3 | 2.014 | -3.325 | 0.032 | 0.016 |
| 228 | Fgf18 | 1.219 | -1.448 | 0.003 | 0.031 |
| 229 | Itgb3 | 2.285 | -2.046 | 0.000 | 0.029 |
| 230 | Haspin | 1.701 | -1.384 | 0.000 | 0.046 |
| 231 | RGD1559482 | 7.425 | -4.108 | 0.000 | 0.048 |
| 232 | Smagp | 2.645 | -1.679 | 0.000 | 0.044 |
| 233 | Rpl7a | 1.332 | -1.220 | 0.000 | 0.032 |
| 234 | Map2k3 | 2.288 | -1.376 | 0.000 | 0.045 |
| 235 | Tnfaip6 | 3.405 | -2.333 | 0.000 | 0.023 |
| 236 | Pdlim4 | 1.919 | -1.470 | 0.000 | 0.011 |
| 237 | AABR07038983.1 | 5.839 | -3.244 | 0.000 | 0.029 |
| 238 | Parvg | 2.754 | -1.736 | 0.000 | 0.046 |
| 239 | AABR07030791.1 | 4.485 | -4.963 | 0.014 | 0.050 |
| 240 | Lilrb3a | 3.913 | -3.356 | 0.000 | 0.011 |
| 241 | Slco4a1 | 1.206 | -1.286 | 0.003 | 0.031 |
| 242 | Lilrb2 | 3.292 | -2.813 | 0.000 | 0.044 |
| 243 | Aoah | 3.940 | -2.992 | 0.000 | 0.012 |
| 244 | Gpx2 | 3.051 | -2.957 | 0.000 | 0.011 |
| 245 | Olr1 | 4.442 | -2.793 | 0.000 | 0.029 |
| 246 | Piezo1 | 2.107 | -1.589 | 0.000 | 0.046 |
| 247 | Slfn4 | 3.542 | -2.958 | 0.000 | 0.023 |
| 248 | RGD1561730 | 5.287 | -5.757 | 0.008 | 0.044 |
| 249 | Pla1a | 3.265 | -1.687 | 0.000 | 0.026 |
| 250 | Itga5 | 3.465 | -2.275 | 0.000 | 0.046 |
| 251 | AABR07018244.2 | 2.765 | -2.427 | 0.000 | 0.049 |
| 252 | Lilrc2 | 5.801 | -3.781 | 0.000 | 0.046 |
| 253 | Zfp36 | 2.643 | -1.846 | 0.000 | 0.022 |
| 254 | Dhrs9 | 2.067 | -1.686 | 0.000 | 0.048 |
| 255 | AC127076.2 | 3.556 | -5.789 | 0.023 | 0.028 |
| 256 | AABR07001555.1 | 1.059 | -1.549 | 0.010 | 0.034 |
| 257 | Slc10a3 | 1.036 | -1.115 | 0.001 | 0.036 |
| 258 | AC125248.1 | 6.939 | -2.931 | 0.000 | 0.012 |
| 259 | AABR07039446.2 | 2.913 | -2.003 | 0.000 | 0.042 |
| 260 | Tfec | 4.457 | -2.548 | 0.000 | 0.050 |
| 261 | LOC690045 | 4.508 | -2.607 | 0.000 | 0.042 |
| 262 | Igfbp3 | 3.247 | -2.276 | 0.000 | 0.039 |
| 263 | AABR07019209.2 | 1.137 | -1.136 | 0.002 | 0.048 |

**Table S3 HLJD intervention upregulated 172 genes**

| Gene number | Gene name | log2FoldChange | | padj | |
| --- | --- | --- | --- | --- | --- |
|  |  | MCAO/R vs Sham | HLJD-H vs MCAO/R | MCAO/R vs Sham | HLJD-H vs MCAO/R |
| 1 | Prodh1 | -1.747 | 1.859 | 0.000 | 0.016 |
| 2 | Ros1 | -3.553 | 2.333 | 0.000 | 0.026 |
| 3 | Col11a2 | -2.226 | 2.020 | 0.000 | 0.007 |
| 4 | Acacb | -1.142 | 1.297 | 0.000 | 0.018 |
| 5 | Ece2 | -1.303 | 1.260 | 0.001 | 0.027 |
| 6 | Dgkg | -1.639 | 1.877 | 0.000 | 0.033 |
| 7 | Chodl | -1.982 | 2.061 | 0.006 | 0.028 |
| 8 | Cldn1 | -1.409 | 2.505 | 0.044 | 0.045 |
| 9 | Adcy5 | -1.089 | 1.263 | 0.000 | 0.017 |
| 10 | Gabrg1 | -1.348 | 1.094 | 0.000 | 0.034 |
| 11 | Prkg2 | -2.104 | 1.490 | 0.000 | 0.046 |
| 12 | Tp53bp2 | -1.184 | 1.124 | 0.000 | 0.011 |
| 13 | Ush2a | -3.568 | 3.059 | 0.000 | 0.000 |
| 14 | Chrne | -2.453 | 2.367 | 0.000 | 0.014 |
| 15 | Pcdh19 | -1.902 | 1.714 | 0.000 | 0.023 |
| 16 | AABR07037995.1 | -1.022 | 1.197 | 0.006 | 0.028 |
| 17 | Dnah9 | -1.315 | 1.742 | 0.000 | 0.034 |
| 18 | Ddc | -2.215 | 2.169 | 0.001 | 0.044 |
| 19 | Sptlc3 | -6.551 | 4.321 | 0.000 | 0.012 |
| 20 | Itgbl1 | -1.300 | 1.607 | 0.005 | 0.050 |
| 21 | Pak3 | -1.452 | 1.212 | 0.000 | 0.014 |
| 22 | Kcnj16 | -1.692 | 1.861 | 0.008 | 0.042 |
| 23 | AABR07057765.1 | -1.708 | 1.295 | 0.000 | 0.025 |
| 24 | Trhr | -3.239 | 2.927 | 0.000 | 0.021 |
| 25 | Rhbdl3 | -1.390 | 1.531 | 0.000 | 0.004 |
| 26 | Slc6a11 | -2.044 | 1.553 | 0.000 | 0.047 |
| 27 | Bcl11b | -1.584 | 1.417 | 0.000 | 0.016 |
| 28 | Nek10 | -2.014 | 1.984 | 0.000 | 0.014 |
| 29 | Slc26a7 | -2.470 | 2.789 | 0.001 | 0.035 |
| 30 | Kcnk16 | -1.991 | 2.387 | 0.035 | 0.012 |
| 31 | Ryr3 | -1.085 | 1.170 | 0.002 | 0.048 |
| 32 | Adhfe1 | -1.481 | 1.562 | 0.001 | 0.047 |
| 33 | Arhgap36 | -4.822 | 3.936 | 0.001 | 0.025 |
| 34 | Plppr1 | -1.442 | 1.118 | 0.000 | 0.047 |
| 35 | Slc17a8 | -2.530 | 2.211 | 0.000 | 0.035 |
| 36 | Igsf1 | -2.477 | 2.205 | 0.000 | 0.016 |
| 37 | Oprk1 | -2.271 | 2.082 | 0.000 | 0.045 |
| 38 | Tex15 | -1.982 | 2.237 | 0.001 | 0.011 |
| 39 | Plxnc1 | -1.609 | 1.376 | 0.000 | 0.003 |
| 40 | Dact1 | -1.383 | 1.290 | 0.000 | 0.036 |
| 41 | Pdzd3 | -3.323 | 2.952 | 0.000 | 0.014 |
| 42 | Ttll9 | -1.470 | 1.859 | 0.000 | 0.004 |
| 43 | Dach1 | -2.152 | 1.695 | 0.001 | 0.048 |
| 44 | Pcdh17 | -1.310 | 1.347 | 0.000 | 0.000 |
| 45 | Asb4 | -4.070 | 3.863 | 0.000 | 0.023 |
| 46 | Papln | -2.398 | 2.301 | 0.000 | 0.035 |
| 47 | Erich3 | -1.193 | 1.110 | 0.002 | 0.035 |
| 48 | Krt2 | -1.581 | 1.764 | 0.000 | 0.036 |
| 49 | Chd3 | -1.506 | 1.134 | 0.000 | 0.030 |
| 50 | Efnb3 | -2.633 | 2.195 | 0.000 | 0.013 |
| 51 | Lhx9 | -3.854 | 3.968 | 0.001 | 0.044 |
| 52 | Gnal | -2.044 | 1.938 | 0.000 | 0.026 |
| 53 | Gabra5 | -1.663 | 1.712 | 0.000 | 0.035 |
| 54 | Chrna7 | -1.378 | 1.240 | 0.000 | 0.045 |
| 55 | Crb1 | -2.882 | 2.471 | 0.000 | 0.028 |
| 56 | Faahl | -3.663 | 2.752 | 0.000 | 0.039 |
| 57 | Pde10a | -1.292 | 1.464 | 0.000 | 0.030 |
| 58 | Filip1 | -2.473 | 1.795 | 0.000 | 0.011 |
| 59 | Slc35d3 | -1.933 | 2.467 | 0.004 | 0.030 |
| 60 | Lix1 | -2.126 | 1.685 | 0.000 | 0.047 |
| 61 | Zcchc12 | -2.346 | 1.813 | 0.000 | 0.018 |
| 62 | Mctp1 | -1.038 | 1.265 | 0.000 | 0.023 |
| 63 | Hes5 | -3.292 | 2.422 | 0.000 | 0.005 |
| 64 | Erbb4 | -1.467 | 1.330 | 0.000 | 0.023 |
| 65 | Qrfpr | -1.457 | 2.227 | 0.001 | 0.038 |
| 66 | Rfx3 | -1.775 | 1.325 | 0.000 | 0.022 |
| 67 | Mas1 | -2.491 | 1.830 | 0.000 | 0.025 |
| 68 | Necab2 | -2.449 | 2.168 | 0.000 | 0.013 |
| 69 | Slc9a2 | -2.989 | 2.566 | 0.000 | 0.026 |
| 70 | Prkce | -1.520 | 1.144 | 0.000 | 0.025 |
| 71 | Rgs14 | -1.403 | 1.169 | 0.000 | 0.044 |
| 72 | Casq2 | -2.093 | 2.075 | 0.000 | 0.011 |
| 73 | Myo16 | -1.727 | 1.254 | 0.000 | 0.039 |
| 74 | Vwa5b1 | -2.790 | 1.685 | 0.000 | 0.039 |
| 75 | Dner | -1.370 | 1.199 | 0.000 | 0.039 |
| 76 | Plppr4 | -1.518 | 1.147 | 0.000 | 0.036 |
| 77 | Nkx1-2 | -3.517 | 2.632 | 0.000 | 0.043 |
| 78 | Dpyd | -1.929 | 1.598 | 0.000 | 0.016 |
| 79 | Hydin | -1.485 | 1.878 | 0.004 | 0.036 |
| 80 | Igfbp5 | -1.732 | 1.763 | 0.002 | 0.044 |
| 81 | Wnt6 | -2.485 | 1.912 | 0.000 | 0.023 |
| 82 | Ca12 | -1.576 | 2.050 | 0.005 | 0.043 |
| 83 | Oprm1 | -1.824 | 1.912 | 0.000 | 0.011 |
| 84 | Slc22a6 | -2.454 | 1.849 | 0.000 | 0.046 |
| 85 | NEWGENE_2134 | -2.761 | 1.901 | 0.000 | 0.033 |
| 86 | Agt | -2.641 | 2.434 | 0.000 | 0.012 |
| 87 | C1qtnf3 | -2.453 | 2.865 | 0.000 | 0.011 |
| 88 | RGD1559896 | -1.410 | 1.015 | 0.000 | 0.021 |
| 89 | Iqca | -2.175 | 1.821 | 0.000 | 0.018 |
| 90 | Syt9 | -2.851 | 2.561 | 0.000 | 0.000 |
| 91 | Kcna5 | -1.885 | 1.803 | 0.001 | 0.030 |
| 92 | Gng7 | -1.366 | 2.101 | 0.000 | 0.017 |
| 93 | Igf2 | -2.217 | 2.108 | 0.000 | 0.014 |
| 94 | Icam5 | -1.416 | 1.409 | 0.000 | 0.037 |
| 95 | Cela3b | -4.681 | 4.041 | 0.000 | 0.033 |
| 96 | Iqub | -1.009 | 1.284 | 0.000 | 0.002 |
| 97 | Frem1 | -1.900 | 2.256 | 0.000 | 0.015 |
| 98 | Zdbf2 | -1.028 | 1.038 | 0.000 | 0.005 |
| 99 | AABR07032520.1 | -1.153 | 1.141 | 0.000 | 0.048 |
| 100 | Pde11a | -1.587 | 2.029 | 0.006 | 0.039 |
| 101 | AABR07056633.1 | -1.706 | 2.527 | 0.006 | 0.004 |
| 102 | Tp73 | -3.030 | 3.702 | 0.003 | 0.011 |
| 103 | Vwa3a | -3.113 | 2.544 | 0.000 | 0.011 |
| 104 | Rgs22 | -1.326 | 2.179 | 0.014 | 0.026 |
| 105 | Iqcg | -1.831 | 3.089 | 0.020 | 0.021 |
| 106 | Fam81b | -1.253 | 1.499 | 0.034 | 0.026 |
| 107 | Pcdhga5 | -1.661 | 1.175 | 0.000 | 0.011 |
| 108 | Ccdc187 | -3.505 | 3.202 | 0.001 | 0.016 |
| 109 | Plppr3 | -1.999 | 1.493 | 0.000 | 0.025 |
| 110 | Sp9 | -1.144 | 1.387 | 0.002 | 0.011 |
| 111 | Fat4 | -1.849 | 1.340 | 0.000 | 0.029 |
| 112 | Hmcn1 | -1.073 | 1.012 | 0.000 | 0.042 |
| 113 | Slc9a5 | -1.507 | 1.172 | 0.000 | 0.049 |
| 114 | Shisa6 | -2.127 | 2.126 | 0.000 | 0.008 |
| 115 | AABR07033882.1 | -2.041 | 1.612 | 0.000 | 0.033 |
| 116 | Plxnb1 | -1.522 | 1.328 | 0.000 | 0.035 |
| 117 | Cbs | -1.462 | 1.478 | 0.000 | 0.023 |
| 118 | Spock3 | -1.115 | 1.139 | 0.000 | 0.030 |
| 119 | Pwwp3b | -1.358 | 1.583 | 0.001 | 0.011 |
| 120 | Dgkb | -1.820 | 1.794 | 0.000 | 0.012 |
| 121 | Htr2c | -2.210 | 2.568 | 0.000 | 0.015 |
| 122 | AABR07071287.1 | -2.901 | 4.735 | 0.000 | 0.005 |
| 123 | AABR07058412.1 | -2.294 | 2.000 | 0.000 | 0.026 |
| 124 | Dcc | -2.028 | 1.686 | 0.000 | 0.014 |
| 125 | Ak9 | -1.988 | 3.106 | 0.034 | 0.043 |
| 126 | Stum | -2.149 | 1.665 | 0.000 | 0.044 |
| 127 | Calml4 | -1.989 | 3.287 | 0.019 | 0.028 |
| 128 | Akr1c19 | -1.467 | 1.743 | 0.010 | 0.026 |
| 129 | Ccdc153 | -2.028 | 2.823 | 0.019 | 0.026 |
| 130 | Ankrd63 | -2.822 | 2.522 | 0.000 | 0.030 |
| 131 | Smim17 | -1.300 | 1.202 | 0.000 | 0.030 |
| 132 | Gria1 | -1.658 | 1.538 | 0.000 | 0.007 |
| 133 | C1ql2 | -1.766 | 1.839 | 0.001 | 0.047 |
| 134 | Gng4 | -1.327 | 1.017 | 0.000 | 0.048 |
| 135 | Dcx | -1.380 | 1.179 | 0.000 | 0.035 |
| 136 | Dnah5 | -1.457 | 2.360 | 0.035 | 0.012 |
| 137 | AABR07045621.1 | -1.377 | 1.193 | 0.000 | 0.047 |
| 138 | Glra3 | -4.822 | 3.856 | 0.000 | 0.012 |
| 139 | Gprasp1 | -1.134 | 1.026 | 0.000 | 0.043 |
| 140 | Col25a1 | -2.309 | 2.188 | 0.000 | 0.011 |
| 141 | Fibcd1l1 | -2.358 | 4.068 | 0.034 | 0.024 |
| 142 | AABR07039303.3 | -2.381 | 2.290 | 0.006 | 0.048 |
| 143 | AABR07059679.1 | -1.361 | 1.248 | 0.000 | 0.028 |
| 144 | AABR07070312.1 | -1.896 | 1.663 | 0.000 | 0.036 |
| 145 | Gdf10 | -1.939 | 2.098 | 0.000 | 0.019 |
| 146 | Pnma3 | -1.919 | 1.350 | 0.000 | 0.046 |
| 147 | Cdh4 | -2.012 | 1.092 | 0.000 | 0.044 |
| 148 | LOC102553088 | -2.356 | 2.355 | 0.000 | 0.021 |
| 149 | Kcng2 | -1.544 | 2.142 | 0.000 | 0.014 |
| 150 | AC103535.1 | -2.367 | 3.415 | 0.022 | 0.036 |
| 151 | Ppp4r4 | -1.112 | 1.277 | 0.000 | 0.004 |
| 152 | AABR07005593.1 | -1.787 | 1.877 | 0.013 | 0.034 |
| 153 | Acot1 | -1.085 | 1.440 | 0.000 | 0.028 |
| 154 | Prtg | -1.418 | 1.938 | 0.040 | 0.042 |
| 155 | Gpr52 | -1.299 | 1.095 | 0.003 | 0.039 |
| 156 | Lrp2 | -1.214 | 1.815 | 0.002 | 0.033 |
| 157 | Unc13c | -2.153 | 1.626 | 0.000 | 0.044 |
| 158 | AABR07013140.1 | -3.823 | 2.794 | 0.001 | 0.043 |
| 159 | Slc12a3 | -2.014 | 2.491 | 0.007 | 0.025 |
| 160 | Scn3b | -1.309 | 1.074 | 0.000 | 0.023 |
| 161 | Sncg | -2.358 | 2.313 | 0.000 | 0.013 |
| 162 | Asic2 | -1.494 | 1.069 | 0.000 | 0.049 |
| 163 | Pnck | -2.123 | 1.569 | 0.000 | 0.047 |
| 164 | Mroh7 | -1.430 | 1.557 | 0.003 | 0.011 |
| 165 | Lamc3 | -1.819 | 1.329 | 0.000 | 0.044 |
| 166 | Dnah12 | -2.125 | 3.295 | 0.027 | 0.011 |
| 167 | Doc2b | -2.063 | 2.075 | 0.000 | 0.023 |
| 168 | Gpc3 | -2.218 | 2.157 | 0.000 | 0.026 |
| 169 | AABR07062799.2 | -1.768 | 1.251 | 0.000 | 0.031 |
| 170 | Lmo7 | -1.374 | 1.099 | 0.000 | 0.042 |
| 171 | AABR07001432.2 | -1.383 | 1.800 | 0.033 | 0.021 |
| 172 | Crym | -1.609 | 1.679 | 0.000 | 0.007 |
